# Adiponectin and the steatosis marker Chi3L1 decrease following switch to raltegravir compared to continued PI/NNRTI-based antiretroviral therapy

**DOI:** 10.1371/journal.pone.0196395

**Published:** 2018-05-10

**Authors:** Obiageli Offor, Netanya Utay, David Reynoso, Anoma Somasunderam, Judith Currier, Jordan Lake

**Affiliations:** 1 Department of Epidemiology, University of Texas Health Science Center Houston, Texas, United States of America; 2 Department of Internal Medicine, University of Texas Health Science Center Houston, Texas, United States of America; 3 Department of Infectious Disease, University of Texas Medical Branch Galveston, Texas, United States of America; 4 Department of Infectious Disease, University of California Los Angeles, California, United States of America; 5 Department of Infectious Disease, University of Texas Health Science Center Houston, Texas, United States of America; Azienda Ospedaliera Universitaria di Perugia, ITALY

## Abstract

**Background:**

People with HIV are at for metabolic syndrome (MetS) and fatty liver disease, but the role of Antiretroviral therapy (ART) is poorly understood. MetS and fatty liver disease been associated with changes in adiponectin, soluble ST2 (sST2), chitinase 3-like 1 (Chi3L1), hyaluronic acid (HA), tissue inhibitor of metalloproteinase-1 (TIMP-1), lysyl oxidase-like-2 (LOXL2) and transforming growth factor β (TGF-β) concentrations in HIV-uninfected populations. Protease (PI) and non-nucleoside reverse transcriptase inhibitors (NNRTI) may contribute to these comorbidities, but the effects of switching from PI- or NNRTI to raltegravir (RAL) on these biomarkers is unknown.

**Methods:**

Cryopreserved plasma was obtained from a completed, prospective trial of HIV-infected women with central adiposity on NNRTI- or PI-based ART during which they were randomized to remain on their current ART or switch to a RAL based regimen. Biomarker concentrations were quantified using ELISA and Multiplex assays at baseline and 24 weeks after randomization. Wilcoxon-signed rank test evaluated within-group changes, Spearman and linear regression models evaluated correlations between biomarkers and clinical covariates.

**Results:**

Participants had a median age of 43 years, CD4^+^ T lymphocyte count 558 cells/mm^3^ and BMI 32 kg/m^2^; 35% met criteria for MetS. At baseline, higher adiponectin levels correlated with higher Chi3L1 levels (r = 0.42, p = 0.02), as did declines after 24 weeks (r = 0.40, p = 0.03). Changes in sST2 correlated with changes in Chi3L1 (r = 0.43, p = 0.02) and adiponectin (r = 0.40, p = 0.03). Adiponectin and Chi3L1 levels decreased significantly in women switched to RAL vs continue PI/NNRTI.

**Conclusion:**

In women with HIV and central obesity, the hepatic steatosis/fibrosis marker Chi3L1 and adiponectin decrease in conjunction with sST2 decreases following switch to RAL. Whether switching from NNRTI/PI-based regimens to RAL can improve hepatic steatosis and dysmetabolism requires further study.

**Trial registration:**

Clinicaltrials.gov NCT00656175

## Introduction

Antiretroviral therapy (ART) has led to a decline in HIV-associated mortality, however as people live longer with treated HIV infection there has been an increase in the prevalence of chronic co-morbidities [1]. Our understanding of the individual contributions of HIV-1 infection, systemic inflammation, and/or immune deficiency remain incomplete. [2]. Despite this, persons with HIV infection on ART appear to be at risk for metabolic and liver disease [3–7].

Liver pathology in HIV-infected patients on ART ranges from steatosis and steatohepatitis to fibrosis, cirrhosis and end stage liver disease [3, 5, 7–9]. The spectrum of associated metabolic derangements is not yet fully understood, but includes adipose tissue dysfunction, dyslipidemia, insulin resistance and the metabolic syndrome (MetS) [1, 7, 8, 10]. There is considerable evidence that ART plays a role in these metabolic derangements, with nucleoside reverse transcriptase inhibitors (NRTI) and protease inhibitors (PI) most commonly implicated in metabolic disruptions [7, 11].

Novel, non-invasive diagnostic procedures to monitor the evolution of liver and metabolic pathology are needed. Circulating biomarkers have the potential to predict and reflect end-organ metabolic changes caused by ART and HIV, but are in need of further exploration. For example, decreased concentrations of the adipokine adiponectin has been linked to MetS, insulin resistance and non-alcoholic liver disease (NAFLD) [12–14]. Adiponectin is an insulin-sensitizing hormone secreted by adipocytes, and unlike other adipokines, adiponectin levels are reduced in insulin resistance, type 2 diabetes mellitus (T2DM) and lipodystrophy [4]. Additionally, hyperglycemia, dyslipidemia and the pro-inflammatory state of MetS [14] are plausible stimuli for the synthesis and release of tissue inhibitor of metalloproteinase (TIMP)-1 [15,16]. Changes in circulating levels of hyaluronic acid (HA), transforming growth factor (TGF)-β, chitinase 3-like (Chi3L1, also known as YKL40), lysyl oxidase-like 2 (LOXL2), and soluble ST2 (sST2) have been observed in obesity, insulin resistance, MetS and liver disease in HIV-uninfected persons, and may be useful biomarkers to detect and monitor these co-morbidities [17–22].

TGF-β_1_ activates hepatic stellate cells to increase extracellular matrix deposition and fibrogenesis [23]. It has many functions including increasing TIMP-1 expression, which inhibits the activity of metalloproteinases that breakdown extracellular matrix [24]. HA is a high molecular weight glycosaminoglycan that is normally synthesized by hepatic Ito cells, deposited in the extracellular matrix, and degraded by sinusoidal endothelial cells [25]. Injury affecting sinusoidal endothelial cells and increased portal pressure leads to accumulation of HA, which has been shown to correlate with severity of inflammation and fibrosis [25–27]. Chi3L1 is a glycoprotein that plays a role in cell proliferation and differentiation, inflammation, and extracellular matrix remodeling by exerting growth factor activity on cells involved in matrix remodeling [28]. Elevated levels of Chi3L1 correlate with greater fibrosis by Ishak and FIB-4 scores [29]. LOXL2 belongs to a family of copper-dependent amine oxidases and specifically promotes fibrotic matrix crosslinking and stabilization [30]. Selective LOXL2 monoclonal antibody blockers suppress the progression of fibrosis and promote fibrosis reversal [30]. sST2 functions as a decoy receptor for IL-33, preventing the pro-inflammatory and pro-fibrotic effects of IL-33 on hepatic stellate cells [31, 32].

We measured concentrations of putative circulating biomarkers of metabolic disease from the cryopreserved plasma of HIV-1 infected women with central obesity on PI-/NNRTI-based ART who were enrolled in the Women, Integrase and Fat Accumulation Trial (WiFAT), a trial of continued NRTI backbone with randomization to switch the 3^rd^ agent to raltegravir (RAL) immediately or continue PI or NNRTI.

## Methods

### Study population

Full methods from the parent WiFAT study have previously been published [10]. Briefly, participants were recruited from five centers in North America between September 2008 and July 2010. Inclusion criteria included age ≥18 years, documented HIV-1 infection, continuous virological suppression since ART initiation, central obesity (waist circumference >94 cm or waist to hip ratio >0.88) [33], HIV-1 RNA <50 copies/mL in the 6 months preceding study entry, ART regimen of a NRTI backbone of tenofovir or abacavir plus emtricitabine or lamivudine plus either a PI or NNRTI, no change in ART in the preceding 12 weeks, and ability and willingness to provide informed consent.

### Study design

Participants were randomized 1:1 to immediate (week 0) or delayed (week 24) switch to open label RAL 400mg orally twice daily. The delayed switch group served as an internal control of continued PI or NNRTI therapy during weeks 0 to 24, with all participants on RAL during weeks 24 to 48. The entry NRTI backbone was maintained throughout the 48 weeks of the study. The parent trial hypothesized that switching from a PI/NNRTI to RAL would be associated with a reversal of central adiposity or impediment to further fat gain. Results showed a 5.4% (p = 0.43) between group difference in visceral fat at 24 weeks using computed tomography-quantified fat area [10].

In this exploratory analysis, measurement of circulating concentrations of biomarkers associated with liver steatosis and fibrosis and MetS were performed on cryopreserved plasma samples obtained from the parent trial. Analyses were performed only for participants with sufficiently remaining cryopreserved plasma samples. The primary endpoint for these analyses was the within-group change in biomarker concentrations 24 weeks after switching to RAL vs continued PI/NNRTI. Institutional review boards and ethics committees of the office of Human Research Protection, University of California, Los Angeles approved all study protocols. Written informed consent was obtained from all participants prior to the initiation of study procedures. The WIFAT study is registered at ClinicalTrials.gov, registration number: NCT00656175.

### Biomarker assessments

Cryopreserved EDTA plasma was obtained from the parent WIFAT study that analyzed fasting (≥8 hours) concentrations of plasma biomarkers. Fourteen participants from the RAL group and 17 from the NNRTI/PI group had sufficient plasma for analysis. Adiponectin, HA and LOXL2 were quantified with ELISA assay, and TGF-β, sST2, Chi3L1 and TIMP-1 were quantified by Luminex assay (all R&D Systems, Minneapolis, MN).

### Statistical analyses

Baseline characteristics between the treatment groups were compared using the Mann-Whitney U-test and Fisher’s exact test for continuous and categorical variables, respectively. Median values and interquartile ranges (IQR) are reported for continuous variables, and percentages for categorical variables. Differences in median changes in biomarker concentrations at baseline and 24 weeks were assessed using the Wilcoxon sign-rank and Mann-Whitney tests. Spearman correlation coefficients of changes in biomarkers, and changes between biomarkers and clinical covariates were calculated. All analyses were conducted as-treated, excluding those participants who did not adhere to study regimen and/or did not have an observed primary endpoint.

All statistical tests were exploratory, with no adjustment for multiple testing. Significance was determined using a two-sided nominal alpha level of 0.05. Bivariate regression modeling was also performed to further assess associations. Data analysis and management were performed using SAS version 9.2 and 9.3 (SAS Institute, Inc., Cary, NC).

## Results

### Patient population

**Fig 1:**
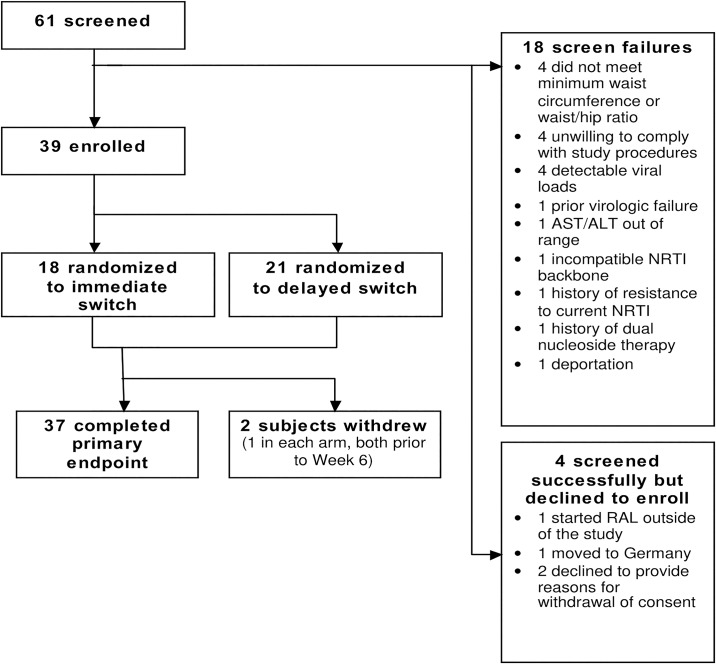
Participant enrollment and disposition.

Participant enrollment and disposition for this trial and the parent trial is shown on Fig 1. Table 1 shows the baseline characteristics of the 37 participants comprising the sample for these analyses. The randomization groups were fairly similar in baseline characteristics, except that the delayed switch group had a higher rate of current tobacco use (60%) compared to the immediate switch group (24%). The median age was 43 years, median body mass index (BMI) 32 kg/m^2^, 59% of participants self-identified as African American, and 16% as Hispanic. Sixty-two percent were on a PI at entry, and 38% were on an NNRTI. The most commonly used NRTIs were tenofovir (78%) and emtricitabine (68%). Based on exclusion criteria, no participants with a diagnosis of diabetes mellitus were enrolled.

**Table 1:** Baseline patient characteristics.

	Immediate Switch*	Delayed Switch*	Overall*
	n = 17	n = 20	n = 37
**Ethnicity**			
**African American**	53%	65%	59%
**Hispanic**	23%	10%	16%
**Age (years)**	41 (39, 47)	46 (36, 51)	43 (37, 49)
**Weight (kg)**	88.7 (81.0, 105.0)	77.7 (71.7, 97.0)	81.8 (73.9, 105.0)
**BMI (kg/m^2^)**	34.7 (28.8, 37.6)	30.4 (27.7, 35.4)	32.0 (28.0, 36.5)
**Tobacco use (current)**	24%	60%	43%
**CD4+ T lymphocyte count (cells/μL)**	563 (447, 747)	554 (354, 770)	558 (422, 747)
**Time on ART (years)**	5.1 (3.1, 7.1)	2.7 (1.6, 6.3)	3.7 (2.4, 7.1)
**ART**			
**PI**	65%	60%	62%
**NNRTI**	35%	40%	38%
**Abacavir**	18%	25%	22%
**Tenofovir**	82%	75%	78%
**VAT (cm^2^)**	145 (105, 154)	137 (93, 154)	138 (100, 154)
**SAT (cm^2^)**	450 (381, 687)	420 (342, 587)	432 (343, 605)
**Diabetes mellitus**֓	0%	0%	0%
**Hyperlipidemia** ֓	18%	25%	22%
**Glucose (mg/dL)**	84 (78, 93)	87 (79, 98)	87 (78, 94)
**Total cholesterol (mg/dL)**	179 (162, 206)	199 (173, 223)	193 (165, 216)
**Triglycerides (mg/dL)**	116 (85, 144)	123 (101, 176)	117 (91, 153)
**LDL (mg/dL)**	113 (103, 123)	116 (93, 142)	116 (94, 130)
**HDL (mg/dL)**	48 (40, 57)	49 (39, 57)	49 (40, 57)

*Percent or median with interquartile range

^֓^ Defined as self-reported diagnosis or on therapy at baseline

BMI: Body mass index. ART: antiretroviral therapy. PI: protease inhibitor. NNRTI: non-nucleoside reverse transcriptase inhibitor. VAT: Visceral adipose tissue. SAT: Subcutaneous adipose tissue. LDL: Low-density lipoprotein cholesterol. HDL: High-density lipoprotein cholesterol.

### Baseline biomarker concentrations

At baseline, no significant differences were observed between the immediate and delayed switch groups for any of the measured biomarkers (Table 2).

**Table 2:** Biomarker levels at baseline and 24 weeks after switch to RAL.

	Immediate Switch (n = 14)				Delayed Switch (n = 17)			
	Median(IQR)				Median (IQR)			
	Week 0	Week 24	Median Δ	p (within group)	Week 0p (between group)	Week 24	Median Δ	p(within group)
**HA(ng/mL)**	48.7(23.2, 81.7)	41.2(26.2, 66.4)	-2.3	0.40	47.5(28.8, 67.7)p = 0.85	36.1(22.2, 63.1)	-2.2	0.52
**TGF-β1(pg/mL)**	31204(13615, 37653)	22942 (11421, 36255)	-3561	0.40	31863(21401, 42534) p = 0.37	27200(15446, 39166)	-1258	0.33
**TGF-β2(pg/mL)**	1663 (1463, 1801)	1576(1385, 1820)	-18	0.36	1750(1526, 1802)p = 0.54	1651(1396, 1783)	-49	0.12
**TGF-β3(pg/mL)**	957(408, 1140)	681(456, 1040)	-61	0.19	100(764.9, 1152) p = 0.55	892(466.6, 1055)	-4.9	0.24
**sST2 (pg/mL)**	12785(8125, 15838)	10047(7826, 12424)	-1202	0.10	9792(7383, 13965)p = 0.35	10146(8083, 11903)	354	0.85
**Chi3L1(pg/mL)**	40166(22752, 54073)	23402(22069, 39897)	-9747	**0.03**	39026(22539, 60770)p = 0.97	30105(18705, 63513)	-5559	0.13
**TIMP-1(pg/mL)**	50588(45517, 57075)	50407(44823, 55746)	-354	0.71	50746(42257, 57211)p = 0.76	48543(43086, 54250)	1908	0.46
**LOXL2(ng/mL)**	0.11(0, 3.4)	0(0, 4.3)	0	0.81	0(0, 1.339)p = 0.38	0(0, 1.1)	0	0.94
**Adiponectin (ng/mL)**	2909(1142, 5183)	1610(935, 4217)	-872	**0.02**	2093(1041, 3232) p = 0.30	1802(1003, 3455)	163	0.49

IQR: Interquartile range, HA: Hyaluronic acid, TGF: Transforming growth factor, sST2: Soluble ST2, Chi3L1: Chitinase 3-Like 1, TIMP-1: Tissue Inhibitor of metalloproteinase 1, LOXL2: Lysyl oxidase-like 2

Δ = difference in plasma biomarker level (Week 24- Week 0).

### Twenty-four-week changes in biomarkers

**Fig 2:**
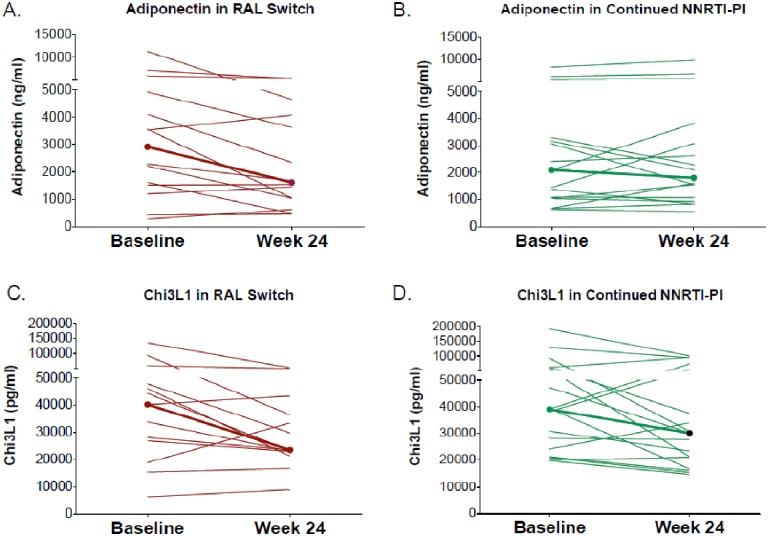
Biomarker changes following switch to RAL vs continued PI/NNRTI.

Changes in biomarkers within each treatment group are shown in Table 2. Between weeks 0 and 24, there was a significant decrease in Chi3L1 in RAL-treated participants (-9747pg/ml; -24%; p = 0.03) but not for those participants that remained on a NNRTI or PI (-5559 pg/ml; -14%; p = 0.13). A significant decrease in adiponectin was also observed among RAL-treated participants (-872 ng/ml, -30%, p = 0.02) with a non-significant increase in adiponectin levels in participants remaining on an NNRTI or PI (163 ng/ml, +7.6%, p = 0.49). No statistically significant within-group changes were observed in the other measured biomarkers between week 0 and 24. Only the changes in adiponectin were significant between groups (P = 0.02) (Table 3). Fig 2a–2d illustrate the 24- week changes from baseline in adiponectin and Chi3L1 within each randomization group.

**Table 3:** Between-group differences in net changes in biomarker levels at 24 weeks.

	Raltegravir (n = 14)	NNRTI/PI (n = 17)		
	Median change (Week 24 minus Week 0)		Median Δ	p- value
**HA (ng/mL)**	-2.307 (-18.2, 6.13)	-2.242 (-23.26, 6.94)	0.06547	0.89
**TGF-β1 (pg/mL)**	-3561 (-7119, 3914)	-1258 (-14021, 5634)	2303	0.77
**TGF- β2 (pg/mL)**	-17.98 (-324, 151.4)	-48.71 (-295.3, 93.39)	-30.73	0.77
**TGF- β3 (pg/mL)**	-60.99 (-329.9, 64.59)	-4.93 (-383.4, 98.87)	56.06	0.98
**sST2 (pg/ml)**	-1201 (-3580, 613.6)	353.6 (-2786, 1583)	1555	0.34
**Chi3L1 (pg/ml)**	-9747 (-23079, 2020)	-5559 (-28072, 5412)	4188	0.93
**TIMP-1 (pg/ml)**	-353.8 (-4404, 4728)	-1908 (-4610, 3952)	-1554	0.49
**LOXL2 (ng/ml)**	0 (-0.1243, 0)	0 (-0.04143, 0)	0	0.59
**Adiponectin (ng/ml)**	-872.1 (-1759, 89.84)	162.8 (-436, 744.2)	1035	0.02

### Correlations between changes in biomarkers and clinical covariates

**Fig 3:**
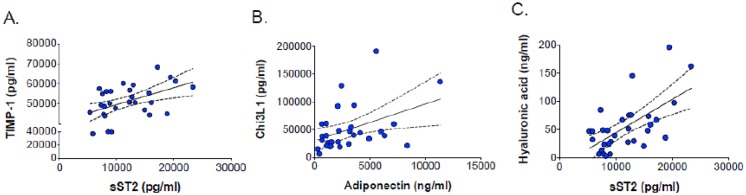
Biomarker correlations at baseline.

**Fig 4:**
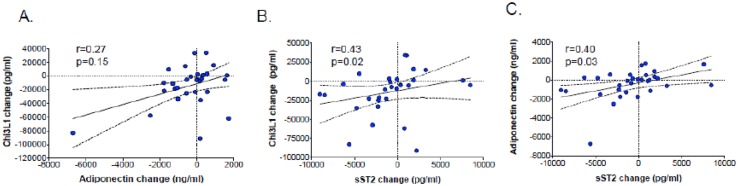
Correlations of 24-week changes in adiponectin, sST2 and Chi3L1.

**Fig 5:**
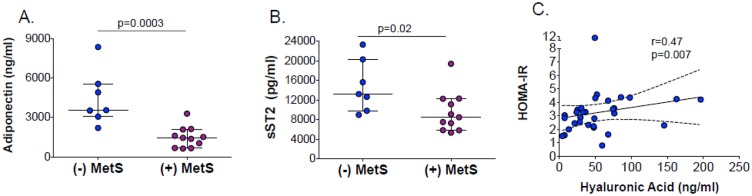
Baseline correlations between biomarker levels & clinical covariates.

At baseline, there were significant positive correlations between TIMP-1 and sST2 (r = 0.48, p = 0.006) (Fig 3a), Chi3L1 and adiponectin (r = 0.42, p = 0.02) (Fig 3b), and between HA and sST2 (r = 0.52, p = 0.003) (Fig 3c). Significant baseline correlations were also observed between high-density lipoprotein (HDL) cholesterol and adiponectin (r = 0.55, p = 0.001) and sST2 (r = 0.38, p = 0.03) concentrations. Higher baseline adiponectin and sST2 concentrations were associated with higher HDL cholesterol levels (r = 0.55, p = 0.001 and r = 0.38, p = 0.03, respectively). Statistically significant negative correlations at baseline were seen between CRP and HA (r = -0.43, p = 0.02), sST2 (r = -0.57, p = 0.001), and TIMP-1 (r = -0.53, p = 0.002). There was a non-significant positive correlation between 24-week changes in adiponectin and Chi3L1 levels (r = 0.27, p = 0.15) (Fig 4a). Significant positive correlations were seen in 24-week changes between sST2 and Chi3L1 (r = 0.43, p = 0.017) (Fig 4b) and between sST2 and adiponectin (r = 0.40, p = 0.03) (Fig 4c). Adiponectin levels were significantly lower in participants with MetS compared to those without MetS (Fig 5a and 5b). Only HA levels correlated with insulin resistance (r = 0.47, p = 0.007), as quantified by the homeostatic model assessment (HOMA-IR) (Fig 5c).

Upon stratification by randomization group, there was a significant correlation between 24-week changes in Chi3L1 and HDL (r = -0.59, p = 0.04) in the RAL group that was not seen in the PI/NNRTI group. No other significant correlations were observed for within-group changes in biomarkers. When data from randomization arms was pooled, there were significant correlations in the 24-week changes between Chi3L1 and sST2 (r = 0.43, p = 0.02), and between adiponectin and sST2 (r = 0.40, p = 0.03). There was a moderate correlation between the 24-week changes in sST2 and HDL cholesterol (r = 0.40, p = 0.03).

## Discussion

In this randomized trial of HIV-infected women with central adiposity, switching to RAL-based ART was associated with a statistically significant decline in adiponectin and Chi3L1. Adiponectin is one of the most studied adipocytokines, and is associated with diseases of adipose tissue dysfunction. While some studies have shown adiponectin to be positively correlated with total body fat, HDL and insulin levels, others suggest that adiponectin levels are reduced in people with insulin resistance, T2DM and lipodystrophy [4, 34–35]. We observed a significant decline in adiponectin levels after switching to RAL-based ART. Based on findings of hypoadiponectinemia in HIV-infected men with lipoatrophy [35–36], a decline in adiponectin after RAL switch is not in keeping with our hypothesis that an ART regimen with an improved metabolic profile would result in higher levels of adiponectin [37]. However, while adiponectin is produced mainly by adipocytes in white adipose tissue, it can also be made by hepatocytes, myocytes, and epithelial cells [38], and circulates both as an inactive low molecular weight form and the bioactive high molecular weight form. The assay used did not distinguish between these forms, so the biological implications of the change in levels are unclear, and the tissue source of circulating adiponectin is unknown. Further studies are needed to understand the mechanism and metabolic effects of RAL on circulating levels of adiponectin.

We also observed a statistically significant decline in Chi3L1 levels in the immediate switch group compared to those who remained on a PI-/NNRTI-based ART regimen. Chi3L1 promotes extracellular matrix deposition and remodeling, with higher Chi3L1 levels correlating with higher Ishak fibrosis scores [29]. The significant decline in Chi3L1 after switching to RAL-based ART suggests that RAL may be associated with a reduced pro-fibrotic milieu compared to PI-/NNRTI-based regimens [28–29]. Elevated levels of Chi3L1 have also been observed in patients with T2DM [39]. In a prospective study of patients with T2DM, all-cause mortality was increased in patients within the second and third tertile of Chi3L1 levels (Hazard ratio (HR) 1.50, p = 0.034 and 2.88, p<0.001, respectively compared with the first tertile) [40]. Although diminished, this association persisted after adjusting for cardiovascular risk factors which have a documented relationship with Chi3L1, and glomerular filtration rate. Although a few studies have identified correlations between Chi3L1 and insulin resistance, dyslipidemia, and acute infections, much remains unknown about the mechanism of Chi3L1 in inflammation and metabolic homeostasis [41–43], particularly in the setting of HIV/AIDS.

In our study, a high baseline concentration of sST2 was associated with higher HDL, TIMP-1, and HA levels and lower hs-CRP levels, and decreased in conjunction with adiponectin and Chi3L1, suggesting that high sST2 levels may reflect a pro-fibrotic rather than pro-inflammatory state. sST2 functions as a decoy receptor for IL-33 in both transmembrane (ST2L) and soluble (sST2) forms [31]. IL-33 is an IL-1 related cytokine that is released in response to cell death and induces a Th2 cytokine response, as occurs during severe hepatic inflammation and fibrosis [31]. Pascual-Figal et al. demonstrated that while IL-33 interaction with the transmembrane receptor showed cardioprotective effects in experimental models, interaction with the soluble decoy receptor sST2 resulted in increased myocardial fibrosis, cardiomyocyte hypertrophy and myocardial dysfunction [44]. It is unclear what common stimulus might be driving both HDL and sST2 production. Nonetheless, sST2 may serve as a surrogate marker for fibrosis and atherosclerosis, components of insulin resistance, metabolic syndrome and diabetes.

When compared to many PIs and NNRTIs, RAL-based ART regimens have a more favorable metabolic profile and have been suggested as an alternative in patients with metabolic disturbances on other regimens [45–47]. Studies analyzing metabolic changes after switching PI-/NNRTI-based ART to RAL-based regimens have used a variety of markers and clinical variables such as BMI, fasting lipid profile, total body fat, visceral adipose tissue quantity and subcutaneous adipose tissue quantity to arrive at their conclusions [46–49]. Domingo et al studied mitochondrial DNA and gene transcripts for PPAR-γ, adiponectin, cytochrome b and TNF-α in patients with HIV-associated lipodystrophy syndrome switching from stavudine to RAL [49], and demonstrated that switching to RAL improved adipocyte differentiation and mitochondrial function in SAT. As our study was not designed to formally assess the utility of the biomarkers measured as indicators of hepatic steatosis, insulin resistance and metabolic syndrome in HIV patients on ART, these relationships require further investigation.

This study has several limitations. It is small in sample size and is a post-hoc, exploratory analysis of biomarkers. The prevalence of obesity in both randomization groups of women in this study (median BMI 32 kg/m^2^) is high, and the results therefore may not be generalizable to the non-obese and male populations. As these are post-hoc exploratory analyses, the lack of a clinical metabolic assessment with which to compare biomarker changes, such as quantification of hepatic steatosis, is also a limitation. Another limitation to our study is the lack of formal assessment of menopausal status by hormonal assessment or self report in the study participants during follow-up. However, early analyses within the parent protocol did not suggest any significant differences in treatment response when stratified using age <50 vs ≥50 as a surrogate for menopause. Participants were not required to keep food diaries during the study period, and no dietary information was obtained either prospectively or retrospectively. Although all biomarker analyses were performed on performed on cryopreserved plasma obtained after ≥ 8 hours of fasting, the lack of dietary data is a limitation. However, this is the first study to our knowledge that has studied the changes in these biomarkers of steatosis and fibrosis after switching from PI/NNRTI to RAL-based ART.

## Conclusion

In women with HIV and central obesity, the adipocytokine adiponectin and Chi3L1, a marker of liver steatosis and metabolic syndrome, decreased significantly following switch to RAL, but not with continued PI or NNRTI. Larger studies are needed to confirm these findings, understand the mechanism of this decline, and determine relationships between these biomarkers and clinical endpoints.

## Supporting information

S1 File CONSORT checklist. (PDF)

S2 File Parent study protocol. (PDF)
