# Supplementary material for: Adiponectin and the steatosis marker Chi3L1 decrease following switch to raltegravir compared to continued PI/NNRTI-based antiretroviral therapy
Source: PLoS One. 2018 May 10;13(5):e0196395. doi: 10.1371/journal.pone.0196395 (PMC5944924; doi:10.1371/journal.pone.0196395)
Supplement: S2 File — (PDF) [file pone.0196395.s002.pdf]

Study Title:

Phase II Study of Raltegravir as Replacement for PI- or NNRTI-Based ART in Women with Fat Accumulation (CARE 001)

Final Version 6.0 dated 06/05/09
Pharmaceutical Support and Study Drug Provided by:
Merck & Co., Inc.

Study Drug:
Raltegravir, Isentress, MK-0518

Protocol Co-Chairs:

Judith Currier, M.D.
Jordan Lake, M.D.
Grace McComsey, M.D.

TABLE OF CONTENTS

PROTOCOL SIGNATURE FORM **4**

APPENDICES **5**

PROTOCOL ROSTER **6**

1.0 STUDY HYPOTHESIS AND OBJECTIVES: **8**

1.1 HYPOTHESIS 8
1.2 PRIMARY OBJECTIVES: 8
1.3 SECONDARY OBJECTIVES: 9

2.0 INTRODUCTION **9**

2.1 BACKGROUND AND RATIONALE 9

3.0 STUDY DESIGN **12**

4.0 SELECTION AND ENROLLMENT OF SUBJECTS **12**

4.1 INCLUSION CRITERIA 12
4.2 EXCLUSION CRITERIA 13

5.0 STUDY TREATMENT **13**

5.1 REGIMENS, ADMINISTRATION, AND DURATION 13
5.2 STUDY PRODUCT FORMULATION AND PREPARATION 14
5.3 PHARMACY: PRODUCT ACQUISITION, DISTRIBUTION, AND ACCOUNTABILITY 14
5.4 CONCOMITANT MEDICATIONS 14

6.0 CLINICAL EVALUATIONS **15**

6.1 SCHEDULE OF EVENTS **15**

6.2 TIMING OF STUDY EVALUATIONS 16
6.5 CLINICAL ASSESSMENTS 17
6.6 LABORATORY EVALUATIONS 19

7.0 TOXICITY MANAGEMENT **20**

7.1. GRADE 1 OR 2 TOXICITY 20
7. 2 GRADE 3 TOXICITY 20
7.3 GRADE 4 TOXICITY 20

8.0 CRITERIA FOR DISCONTINUATION **21**

8.1 PERMANENT STUDY DRUG DISCONTINUATION 21
8.2 PREMATURE STUDY DISCONTINUATION 21
8.3 EARLY DISCONTINUATION 21

9.0 SAMPLE SIZE AND ENDPOINTS **21**

9.1 SAMPLE SIZE 21
9.2 RANDOMIZATION AND STRATIFICATION 22
9.3 PRIMARY ENDPOINT ASSESSMENTS 22
9.4 SECONDARY ENDPOINTS ASSESSMENTS 22
9.5 STUDY MONITORING 22
9.6 ANALYSES 22

10.0 DATA COLLECTION AND MONITORING AND ADVERSE EVENT REPORTING **23**

10.1 RECORDS TO BE KEPT 23
10.2 ROLE OF DATA MANAGEMENT 23
10.3 CLINICAL SITE MONITORING AND RECORD AVAILABILITY 23

10.4 SERIOUS ADVERSE EVENT REPORTING **23**

11.0 HUMAN SUBJECTS **24**

11.1 INSTITUTIONAL REVIEW BOARD (IRB) REVIEW AND INFORMED CONSENT 24
11.2 SUBJECT CONFIDENTIALITY 24
11.3 STUDY DISCONTINUATION 24

12.0 PUBLICATION OF RESEARCH FINDINGS **24**

13.0 BIOHAZARD CONTAINMENT **24**

14.0 PROPOSED SITES **25**

15.0 TIMELINE .... ERROR! BOOKMARK NOT DEFINED.

16.0 REFERENCES **26**

APPENDIX I **28**

APPENDIX III – CLINICAL TRIALS PROTOCOL REGISTRATION # **42**

APPENDIX IV - CONSENT FOR PHOTOGRAPHY

Protocol Signature Form

The signatures of the Site Investigator and Monitor below constitute their approval of this protocol and provide the necessary assurances that this study will be conducted according to all stipulations of the protocol, including all statements regarding confidentiality and in compliance with the current version of the protocol, International Conference on Harmonization Good Clinical Practice E6 (ICH-GCP), and the applicable national and local regulatory requirements.

Investigator's Name	Signature of Investigator	Date

Appendices

**APPENDIX I**	**Data and Safety Monitoring Committee**
**APPENDIX II**	**Model Informed Consent**
**APPENDIX III**	**Clinical Trials Protocol Registration**
**APPENDIX IV**	**Consent for Photography**

Protocol Roster

Protocol Co-Chair
Judith Currier, M.D.
Professor of Medicine
UCLA CARE Center
David Geffen School of Medicine at UCLA
9911 W. Pico Blvd., Suite 980
Los Angeles, CA 90035
P: 310-557-1891
F: 310-557-1899
jscurrier@mednet.ucla.edu

Protocol Co-Chair
Jordan Lake, M.D.
UCLA Division of Infectious Diseases
1399 S. Roxbury Drive
Los Angeles, CA 90035
P: 310-557-2273
F: 310-557-3450
jlake@mednet.ucla.edu

Protocol Co-Chair
Grace McComsey, M.D.
Associate Professor of Pediatrics and Medicine
Case School of Medicine
11100 Euclid Avenue
Cleveland, Ohio 44106
P: 216-844-3645
F: 216-844-8362
mccomsey.grace@clevelandactu.org

UCLA
Maricela Gonzalez Operations Manager
1399 S. Roxbury Drive, Suite 100
Los Angeles, CA 90035
P: 310-557-3743
F: 310-557-3450
mmgonzalez@mednet.ucla.edu

Vanessa Cajahuaringa
Field Representative
1399 S. Roxbury Drive
Los Angeles, CA 90035
P: 310-557-3798
F: 310-557-3450
VCajahuaringa@mednet.ucla.edu

Carmen Malouf
Fund Manager
9911 W. Pico Blvd., Suite 980
Los Angeles, CA 90035
P: 310-557-3615
F: 310-557-1899
cmalouf@mednet.ucla.edu

Faith Landsman
Regulatory Administrator
9911 W. Pico Blvd., Suite 980
Los Angeles, CA 90035
P: 310-557-3675
F: 310-557-1899
Flandsman@mednet.ucla.edu

Heather McCreath
Data Management Center
Division of Geriatrics, UCLA
10940 Wilshire, Suite 900
Los Angeles, CA 90024
P: 310-267-0848
F: 310-267-4257
hmccreath@mednet.ucla.edu

Case Western
Corrilynn Olesky Hileman, M.D.
University Hospitals Case Medical Center
Department of Infectious Diseases
2061 Cornell Road
Foley Building-Room 411/Mailstop 5083
Cleveland, OH 44106
P: 216-844-1632
F: 216-844-5523
Colesky@neoucom.edu

Jane Baum BSN RN, Clinical Coordinator
Case Western Reserve University
University Hospitals Case Medical Center
Clinical Trials Unit
2061 Cornell Road Room 303A
Cleveland, OH 44106
P: 216.844.2546
F: 216.844.5523
Baum.jane@clevelandactu.org

Cheryl Smith

Study Coordinator
Smith.Cheryl@clevelandactu.org

Dawn Antosh
Study Nurse
Antosh.dawn@clevelandactu.org

Tufts University School of Medicine

Christine Wanke, M.D.
Professor of Medicine
Tufts University School of Medicine
150 Harrison Avenue
Boston, MA 02111
P: 617-636-3811
F: 617-636-3810
christine.wanke@tufts.edu

Alexandra Mangili, M.D.
Assistant Professor
P: 617-636-3811
F: 617-636-3810
Alexandra.mangili@tufts.edu

Jul Gerrior, MA, RD, LDN
Research Dietician
Tufts University School of Medicine
Dept. of Public Health & Family Medicine
150 Harrison Avenue, Jaharis 260
Boston, MA 02111
P: 617-636-0492
F: 617-636-38103662
jul.gerrior@tufts.edu

Tufts CT Reading Center

Andrea L. Desilets
Research Coordinator
Body Composition Analysis Center
Tufts University Friedman School of Nutrition Science and Policy
150 Harrison Ave., Jaharis Room #212
Boston, MA 02111
P: 617-636-3497
F: 617-636-3662
Andrea.desilets@tufts.edu

Vanderbilt University

Todd Hulgan, M.D., M.P.H.
Vanderbilt AIDS Clinical Trials Center
345 24th Avenue North, Ste. 105
Nashville, TN 37203
P: 615-467-0154 x105
F: 615-467-0158
todd.hulgan@vanderbilt.edu

Rebecca Basham

Clinical Trials Specialist
Vanderbilt ACTC
345 24th Ave. N., Suite 105
Nashville, TN 37203
P: 615-467-0154 x118
F: 615-467-0158

rebecca.j.basham@Vanderbilt.Edu

University Health Network - Toronto General Hospital, Immunodeficiency Clinic

Sharon Walmsley, M.D.
585 University Ave 13N
Toronto, Ontario M5G 2N2
P: 416-340-38715077
F: 416-340-9105
Sharon.Walmsley@uhn.on.ca

Warmond Chan, RN, CCRP
Clinical Trials Coordinator
585 University Ave.
13 North, Room 1323
Toronto, Ontario
Canada M5G 2N2
P: 416-340-4800 Ext 6954
F: 416-340-9105

Warmond.Chan@uhn.on.ca

Rosemarie J. Clarke RN, CCRP
Senior Clinical Research Supervisor
Tel: 416-340-4800 ext: 6723
Rosemarie.Clarke@uhn.on.ca

1.0 Study Hypothesis and Objectives:

1.1 Hypothesis

Compared to continued treatment with a PI- and/or NNRTI-based regimen, substituting raltegravir will be associated with a 10% reduction in visceral adipose tissue over 24 weeks.

1.2 Primary Objectives:

1.2.1 To compare the visceral adipose tissue volume by abdominal CT scan at 24 weeks between women who switch to a raltegravir-based regimen and those who remain on currently suppressive PI- or NNRTI-based ART.

1.2.2 To describe the safety and tolerability of raltegravir compared to continued treatment among women over the 48 week duration of the study.

1.3 Secondary Objectives:

1.3.1 To compare visceral adipose tissue volume by abdominal CT scan at 48 weeks between women in the immediate-switch versus those in the delayed-switch to raltegravir arms.

1.3.2 To compare the SAT and VAT:SAT ratio in women who switch to a raltegravir-based regimen and those who remain on currently suppressive PI- or NNRTI-based ART at 24 weeks.

1.3.3 To compare Framingham risk scores for women who switch to raltegravir-based ART and those who remain on PI- and/or NNRTI-based ART after 24 and 48 weeks of treatment.

1.3.4 To compare the prevalence of metabolic syndrome in women who switch to raltegravir-based ART and those who remain on PI- and/or NNRTI-based ART at weeks 24 and 48.

1.3.5 To evaluate quality of life for women who switch to raltegravir-based ART and those who remain on PI- and/or NNRTI-based ART.

1.3.6 To compare changes in fasting lipids and HOMA-IR between women who switch to raltegravir-based ART and those who remain on PI- and/or NNRTI-based ART.

1.3.7 To compare changes in hsCRP between women who switch to raltegravir-based ART and those who remain on PI- and/or NNRTI- based ART

2.0 Introduction

2.1 Background and Rationale

The term lipodystrophy has been used to describe all forms of body fat redistribution and metabolic changes, and is not precise (2, 3, 14). Fat abnormalities fall into distinct categories of lipoatrophy and lipohypertrophy, each syndrome with different risk factors, clinical features, and treatments.

Lipohypertrophy is defined by fat accumulation in the abdomen, breasts, and posterior neck (buffalo hump). Risk factors in observational and cross sectional studies have included PI use, older age, white race, more advanced degree of immunosuppression, and female gender. The prevalence of fat redistribution has been reported to range from 2%-84% (3, 7, 14, 16). The wide interval reflects lack of consensus definition and diagnostic criteria. Cross-sectional imaging techniques such as computed tomography

and magnetic resonance imaging are the gold standard for measurement of body fat in the research setting.

The diagnosis of lipohypertrophy used in clinical studies has included a waist to hip ratio (>0.94 in men and >0.88 in women) or enlarged abdominal circumference (>95 cm in men and >94 cm in women) (6). Recent prospective trials in treatment naïve patients have called into question the role of protease inhibitors as a cause of lipohypertrophy (Carr, 9th IWLADR, 2007, study BI82.33). After 48 weeks of therapy with either a tipranavir- or r/lopinavir-based regimen combined with emtricitabine, visceral fat decreased in both groups. It is unknown whether a longer course of treatment with a PI might contribute to the development of lipohypertrophy. In ACTG 5142, EFV-based regimen induced the same level of trunk fat increase as that seen in LPV/RTV arm (9), again questioning the role of PI in the induction of the abdominal fat accumulation seen in subjects with HIV.

The decision to treat fat redistribution is frequently determined in the context of associated findings such as insulin resistance and hyperlipidemia. There is no consensus in regard to whether treatment of body fat changes should be undertaken in the absence of an associated metabolic syndrome.

Changing ART may be appropriate for patients with lipoatrophy, particularly among patients currently receiving stavudine or zidovudine when tenofovir or abacavir are available therapeutic options (12, 13, 15,). A recent study showed that after 48 weeks of therapy with either zidovudine or tenofovir, individuals on zidovudine had clear limb fat losses, while those on tenofovir had increased limb fat. Interpretation of the study is limited by the lack of baseline DEXA testing (1). Another study demonstrated that switching from a thymidine-containing regimen (d4T or AZT) to either tenofovir or abacavir resulted in small but statistically significant gains in limb fat, with most marked improvement seen in patients switching from stavudine-containing regimens (15). Fat losses from stavudine improved, but only approximately 10% of fat recovery was seen at one year follow-up, and it is unclear if longer time intervals will lead to substantial gains, or if these individuals will ever return to pre-treatment baseline.

Recombinant human Growth Hormone (rhGH) has been found to decrease visceral fat, but also exacerbates insulin resistance and worsens lipoatrophy in clinical trials (18). Benefits gained from rhGH appear to be reversed upon cessation of the drug. GH-Releasing Factor has been shown to decrease visceral fat and improve lipid profile, but until final results of phase 3 trials are available, cannot be recommended (6). Metformin has been shown to improve glucose tolerance, reduce weight and visceral fat, and decrease triglyceride levels in randomized trials of HIV-infected individuals with central fat accumulation and insulin resistance. Buffalo hump liposuction has been associated with surgical complications and high recurrence rate, and may not be covered by insurance. Unlike switch therapy for lipoatrophy, switching off PI-based regimens to date has not been shown to improve lipohypertrophy, however most studies have switched from PI to NNRTI (which may have an independent negative effect on body fat) and have not controlled the nucleoside component of therapy.

Central fat accumulation, or lipohypertrophy is a common and distressing problem for women with HIV infection. In addition, the metabolic effects of increased visceral fat may contribute to long-term dyslipidemias, insulin resistance, and increased cardiovascular risk. There are currently no FDA-approved treatments for central fat accumulation for

HIV-infected patients. Although central fat accumulation is usually attributed to antiretrovirals, the contribution of PIs and/or NNRTIs to the development or persistence of this problem remains unclear. Recent data suggests that the thymidine nucleoside analogues are likely to be a major contributor to the development of lipoatrophy and possibly lipohypertrophy, however, the contribution of other classes of drugs (PIs and NNRTIs) to the development of lipohypertrophy is less clear.

Raltegravir has demonstrated potent in vivo activity in treatment-naïve and treatment-experienced HIV-infected subjects. In a trial of treatment-naïve subjects, 197 subjects were randomized to receive one of 4 doses of raltegravir or EFV with tenofovir and emtricitabine (11). At week 24, 85-95% of subjects in all dose groups of raltegravir achieved an HIV-1 RNA level of <50 copies/mL. Raltegravir was generally well-tolerated, with only one subject discontinuing drug due to elevated AST. In 167 treatment-experienced subjects (50% had a baseline phenotypic score of 0 for the drugs available in the optimized background regimen), three doses of raltegravir (200 mg, 400 mg, and 600 mg, each given BID) were evaluated (8). Raltegravir lowered viral load by at least 2 $\log_{10}$ copies/mL in all subjects within 2 weeks after initiating treatment and suppressed viral load to less than 50 copies/mL in 56-72% of subjects within 16 weeks. The most common adverse event (AE) rates in subjects receiving raltegravir were comparable to controls, and included headache, fatigue and dizziness. The dose of 400 mg BID was chosen for further development of raltegravir. In the studies of raltegravir in both treatment-naïve and treatment-experienced subjects, each dose arm had similar efficacy.

Phase III studies of raltegravir have been conducted in multi-drug resistant patients and in treatment naïve patients. BENCHMRK-1 and -2 were conducted in patients with triple class resistance (5, 17). In these studies, raltegravir was compared to placebo each with optimized background therapy. At week 16, subjects receiving raltegravir had significantly higher rates of HIV-1 RNA levels <50 copies/mL (61% in BENCHMRK-1 and 62% in BENCHMRK-2) compared with subjects receiving placebo (33% and 36%). In both trials, raltegravir was generally well-tolerated, with an AE profile similar to that of placebo.

Preliminary data suggest that raltegravir has no appreciable impact on serum lipids at week 24 and this difference is significant when compared to efavirenz (Table). There is currently no data on the impact of raltegravir on body fat changes and limited data on the tolerability of raltegravir among women.

Treatment	Mean BL TC mg/dl	Change from BL at wk 24	Mean BL TG Mg/dl	Change from BL to wk 24
MK 518 400 mg BID n=40	168	-7 (-15 to 2)	127	-2 (-23 to 18)
Efavirenz	170	+19 (8 to 30)	128	+47 (-1 to 96)

Teppler ICAAC 2006, abstract 256a

The goal of this Phase II study is to evaluate the safety and tolerability of a raltegravir-based regimen (without thymidine nucleosides) in virologically suppressed women who are experiencing central fat gain during effective ART.

3.0 Study Design

Phase II Randomized 1:1, open-label study of continued current ART compared to a switch from PI or NNRTI to a raltegravir-based regimen in women with lipohypertrophy and suppressed HIV-1 RNA on stable therapy. All subjects will remain on a stable background regimen of tenofovir or abacavir AND emtricitabine or lamivudine while switching from PI or NNRTI to raltegravir. After 24 weeks, all patients will be switched to a raltegravir-based regimen and continue on observation for an additional 24 weeks.

4.0 Selection and enrollment of subjects

4.1 Inclusion Criteria

4.1.1 HIV positive women age 18 and older.

4.1.2 HIV-1 infection, as documented by any licensed ELISA test kit and confirmed by Western blot at any time prior to study entry or plasma HIV-1 RNA > 2000 on two occasions.

4.1.3 Documented central fat accumulation (defined by waist circumference of > 94 cm or a waist to hip ratio of >0.88).

4.1.4 HIV-1 RNA documented to be less than 50 copies/ml at screening. Patients are required to have had continuous virologic suppression (defined as <500, 400, 75 or 50 copies/ml as indicated by the assay in use at the time) since the initiation of their first ART regimen. Subjects who have experienced transient viremia ("blips") to < 500 copies/ml with subsequent re-suppression will be permitted to enroll.

4.1.5 Current antiretroviral therapy with two nucleoside analogues and either a non-nucleoside analogue (nevirapine, efavirenz, or etravirine) or an approved protease inhibitor. Patients on NNRTI+PI at study entry will be excluded. Study participants are not required to be on their first regimen. However, patients who are not on their first ART regimen must not have previously substituted drugs secondary to virologic failure. Other reasons for substitution such as medication intolerance or patient preference are acceptable. Documentation must be available in the medical record that prior changes in ART were not secondary to virologic failure. No changes in ART in the 12 weeks prior to screening. The nucleoside backbone must include either tenofovir or abacavir and either lamivudine or emtricitabine. Fixed dose combinations with emtricitabine or abacavir are allowed.

For females of reproductive potential (women who have not been post-menopausal for at least 24 consecutive months, i.e., who have had menses within the preceding 24 months, or women who have not undergone surgical sterilization, specifically hysterectomy, bilateral oophorectomy, and/or tubal ligation), will need a negative serum or urine pregnancy test within 48 hours prior to entry. If participating in sexual activity that could lead to pregnancy, study participants must use

contraception while on study drug and for 4 weeks after going off study drug.

4.1.6 Ability and willingness of subject to provide informed consent.

4.2 Exclusion Criteria

4.2.1 Pregnancy (current or within the past 6 months) or nursing.

4.2.2 Current use of metformin or thiazolidinediones.

4.2.3 Use of growth hormone or growth hormone releasing factor in the 6 months prior to screening.

4.2.4 Change or initiation of anti-hyperlipemic regimen in the 3 months prior to randomization. Use of a stable anti-hyperlipemic regimen during the study is allowed.

4.2.5 Current use of androgen therapy.

4.2.6 Intent to significantly modify diet or exercise habits, or to enroll in a weight loss intervention during the study period.

4.2.7 Current or projected need to use rifampin, dilantin, or phenobarbital during the 48-weeks of the study or in the past 14 days.

4.2.8 Screening laboratory values as follows:

ANC < 500 cells/mm3
Hemoglobin <10 gm/dl
Cr Cl < 60 ml/min (estimated by Cockcroft-Gault equation)
AST or ALT > 3 times ULN

4.2.9 Evidence of resistance to any component of a subject's current ART regimen (genotypic or phenotypic).

4.2.10 Prior use of single or dual nucleoside regimens (without a third active agent), or history of any ART that would not be considered highly active by current standards.

5.0 Study Treatment

5.1 Regimens, Administration, and Duration

Study treatment is open label raltegravir.

At entry subjects will be randomized 1:1 by the study central office at UCLA to:

ARM A (immediate switch): Switch PI(s) or NNRTI component of therapy to raltegravir 400 mg BID

or

ARM B (delayed switch): Continue current therapy for 24 weeks, then switch PI or NNRTI to raltegravir

If randomized to ARM A at study entry, the subject will substitute raltegravir 400 mg po BID for the protease inhibitor(s) or NNRTI in their current regimen. If subjects are on T20 or maraviroc, this maybe continued unchanged during the study

The nucleoside backbone will be managed as follows:

-If the subject is currently on tenofovir with lamivudine or emtricitabine this will be continued.

-If the subject is currently on abacavir with lamivudine or emtricitabine this will be continued.

-Subjects on emtricitabine or abacavir at study entry will continue these unchanged.

-Use of Atripla at study entry will be considered equivalent to emtricitabine + efavirenz.

5.2 Study Product Formulation and Preparation

Raltegravir (MK-0518) 400-mg tablets must be dispensed in the original bottle with the desiccant provided. Store at room temperature (25°C, 77°F) and protect from moisture.

5.3 Pharmacy: Product Acquisition, Distribution, and Accountability

Study Product Acquisition/Distribution

Raltegravir will be supplied by Merck and Company. Each study site will receive a supply of raltegravir once IRB approval is obtained.

The nucleoside component of therapy will be continued from the prior regimen (tenofovir or abacavir combined with either lamivudine or emtricitabine).

5.4 Concomitant Medications

Prohibited Medications

The prohibited medications with raltegravir are dilantin, phenobarbitol and rifampin.

6.0 Clinical Evaluations

6.1 Schedule of Events

	Screen	Baseline*	Week 4	Week 8	Week 12	Week 18	Week 24*	Week 28	Week 32	Week 36	Week 42	Week 48*	Week 72*
Targeted physical exam with weight, blood pressure		X	X	X	X	X	X	X	X	X	X	X	X
Complete physical exam and height	X												
Body image /Quality of life questionnaire		X					X					X	X
Depression screening		X					X					X	X
CT- L4/5		X					X					X	X
Mid-waist, hip and neck circumferences and waist/hip ratio	X	X					X					X	X
HIV-1 RNA	X		X	X	X		X	X	X	X		X	X
Safety labs**	X	X	X	X	X	X	X	X	X	X	X	X	X
CD4 cell counts		X			X		X					X	X
Fasting lipids, hsCRP, banked serum and plasma samples for insulin, markers of inflammation		X					X					X	X
Urine samples for markers of oxidant stress and endothelial activation		X					X					X	X

* Entry, week 24, 48, and 72 laboratory evaluations are required to be fasting (at least 8 hours)

**Safety labs include: CBC with differential, chemistry panel including liver enzymes (ALT, AST, Alk Phos) and total bilirubin, serum creatinine, pregnancy test. All safety labs, HIV-1 RNA, and CD4 cell counts will be done at local certified laboratories.

6.2 Timing of Study Evaluations

Visit I Screening
After obtaining informed consent, study participants will undergo a series of screening evaluations to determine eligibility. The waist and hip measurements will be performed first to determine if the subject meets the criteria of either a waist measurement > 94cm or a waist to hip ratio >0.88. All sites will be trained in the procedures for obtaining waist and hip measurements, and these will be collected in triplicate.

If eligible, the subject will undergo a single 5 mm slice CT scan at the level of L4-L5, and this image will be sent to the central reading center in electronic format. The CT scan will not be scheduled until eligibility is confirmed by exam and laboratory parameters.

Visit 2 Baseline (within 30 days of screening)
Once eligibility has been confirmed, the subject will be randomized 1: 1 to either:

ARM A: Immediate switch of PI(s) or NNRTI to raltegravir
ARM B: Continue current therapy unchanged for 24 weeks then switch PI(s) or NNRTI to raltegravir.

Visit windows for all subsequent visits will be +/- 14 days from the expected date.

Visit 12
At this visit subjects will be able to either remain on their current regimen, with raltegravir or they may consult with their primary care provider and discontinue raltegravir if they wish.

Post-intervention Follow-up Visit (Week 72)
A post-intervention visit will occur at Week 72. The goal of this visit is to assess: the longer-term safety of raltegravir, differences in patients continuing raltegravir post-intervention vs. those who choose to resume their previous (or another) ART regimen. The format of this visit will be similar to the Week 48 visit.

6.3 Pregnancy

Subjects who become pregnant after study entry must discontinue study treatment immediately. Subjects will continue to be followed on study/off study treatment as per the Schedule of Events in Section 6.1, except for CT scans. Pregnancies that occur on study should be reported to The Antiretroviral Pregnancy Registry. More information is available at www.apregistry.com.
Phone: 800-259-4263; Fax: 800-800-1052.

6.4 Discontinuation Evaluations
Evaluations for Randomized or Registered Subjects Who Do Not Start Study Treatment
Subjects who do not start study treatment will be taken off study with no further evaluations required. The subject will be replaced. All case report forms (CRFs) must be completed and keyed for the period up to and including week 0.

Premature Study Treatment/Background ART Discontinuation Evaluations
Subjects who discontinue raltegravir due to an AE will be followed. The subject will complete the week 12 and 24 evaluations (as listed in the Schedule of Events (section 6.1)) and then be taken off study.

Subjects who discontinue raltegravir for more than 14 days or who have confirmed virologic failure (two measurements > 50 copies/ml) will complete the week 12 and 24 evaluations, as listed in the Schedule of Events (section 6.1), and then be taken off study.

Subjects who discontinue their background ART entry regimen for ≥14 consecutive days due to toxicity or adherence will complete the week 12 and 24 evaluations, as listed in the Schedule of Events (section 6.1), and then be taken off study.

6.5 Clinical Assessments

Documentation of HIV
HIV-1 infection, as documented by any licensed ELISA test kit and confirmed by Western blot at any time prior to study entry. HIV-1 culture, HIV-1 antigen, plasma HIV-1 RNA, or a second antibody test by a method other than ELISA are acceptable alternative confirmatory tests. This does not need to be recorded on a CRF.

Medical History
The medical history must include all AIDS-related diagnoses and any history of coronary artery disease, hypertension, diabetes, or stroke. For current criteria, refer to the appendix identified in the study CRF. Any allergies to any medications and their formulations must be documented. Surgeries that are planned prior to the time of the subject's informed consent will also be documented. Current and previous smoking history will also be collected.

Medication History
A medication history must be recorded in the source documents and the CRF. The medication history will include the following:

- Complete HIV treatment history, including start and stop dates of any current or past antiretroviral medication (estimated if the exact dates cannot be obtained), immune-based therapy, or HIV-related vaccines, including blinded study medications. For patients who are not on their first ART regimen, documentation must be provided that ART medications were not switched secondary to virologic failure. HIV viral loads following any ART switch should be provided to document persistent virologic suppression post-switch. Any available genotypic or phenotypic information should also be included.
- Complete treatment history of any prescription medications taken for the treatment or prophylaxis of opportunistic infections, including actual or estimated start and stop dates.
- All prescription medications (in addition to those noted above) taken in the 30 days prior to study entry, including actual or estimated start and stop dates.
- Nonprescription medications taken in the 30 days prior to study entry. Include actual or estimated start and stop dates.
- Alternative therapies and dietary supplements taken in the 30 days prior to study entry. Include actual or estimated start and stop dates.

Complete Physical Exam
A complete physical examination is required at screening and includes: examination of the skin, head, mouth, and neck; auscultation of the chest; cardiac exam; abdominal exam; examination of the lower extremities for edema. The exam also includes measurement of mid-waist, neck, and hip circumferences, a waist/hip ratio calculation, and documentation of signs and symptoms,

diagnoses, vital signs (temperature, pulse, respiration rate, and blood pressure), height, and weight. In addition, the exam includes an assessment (and subject reported changes) of neck fat, breast enlargement, lipomas, and peripheral lipoatrophy.

Targeted Physical Exam
A targeted physical examination at pre-entry, entry, and all post-entry visits is to be driven by any previously-identified or new signs or symptoms that the subject has experienced since the last visit. This also includes weight, vital signs (temperature, pulse, respiration rate, and blood pressure), and diagnoses at all visits.

Height
Height in stocking feet will be recorded on the CRF at the screening visit only.

Signs and Symptoms
At entry, record all signs and symptoms occurring in the 30 days prior to entry. After entry, record all signs and symptoms ≥ Grade 2. This study will use the DAIDS toxicity grading system. Any signs or symptoms that led to a change in the study drug raltegravir, regardless of grade, must be recorded.

Quality of Life and Body Image Questionnaire
Study subjects will fill out the Phase V questionnaire at entry, week 24, week 48, and week 72.

Depression Screening
Subjects will be asked to complete the standardized and validated CES-D screening tool at weeks 0, 24, 48, and 72.

Diagnoses
Record all diagnoses identified by the ACTG Criteria for Clinical Events and Other Diseases. All confirmed and probable diagnoses made since the last visit will be recorded.

Concomitant Medications
All concomitant medications taken since the last visit will be recorded in the source documents. Please note that only prescription medications will be recorded on the CRFs.

Antiretroviral Medications
All modifications to antiretroviral medications including initial doses, subject-initiated and/or protocol-mandated interruptions, modifications, and permanent discontinuation will be recorded on the CRFs.

Study Treatment Modifications
Modifications of all ARVs including study treatment, initial dose, subject-initiated, and/or protocol-mandated modifications as well as permanent discontinuation will be recorded on the CRFs. If a subject misses more than 4 consecutive doses, the study treatment is considered modified and this must be reported appropriately on the CRF.

CT Scan

Single-slice CT abdomen at the level of L4/L5, with measurement of visceral and subcutaneous fat, will be read centrally by Tufts University. Please see Manual of Operating Procedures for instructions.

Photography
Subjects will be asked to participate in an optional portion of the study aimed at providing visual documentation of body habitus changes while on raltegravir. Subjects who sign a separate consent for this portion of the study will have their individual sites of lipohypertrophy photographed at 0, 24, and 48 weeks. No photographs of the face will be taken to protect the identity of the subject. A separate optional consent form will be provided for this portion of the study (Appendix IV).

6.6. Laboratory Evaluations

Fasting will be required for study entry and weeks 24, 48, and 72. Fasting will be defined as nothing by mouth except water and medications for at least 8 hours prior to the lab draw. At screening, pre-entry, and entry, all laboratory values, regardless of grade, must be recorded on the CRFs. For post-entry assessments, record all laboratory values ≥ Grade 2. Any laboratory value that led to a change in treatment (raltegravir), regardless of grade, must be recorded. Fasting lab studies include: total cholesterol, LDL cholesterol, triglycerides, HDL cholesterol, glucose, and insulin.

Sites must refer to the Division of AIDS Table for Grading the Severity of Adult and Pediatric Adverse Events (DAIDS AE Grading Table), Version 1.0, December 2004. The table is available on the Raltegravir study website at http://www.uclacarecenter.org/iisp2.org.

Pregnancy Test
For women with reproductive potential: Serum or urine β-HCG (urine test must have a sensitivity of 25-50 mIU/mL) must be performed. Testing must be repeated whenever pregnancy is suspected, in addition to the regularly scheduled evaluations.

Fasting lipids, glucose, insulin, hsCRP
Total cholesterol, HDL cholesterol, LDL cholesterol (direct), triglycerides, hsCRP, and glucose will be performed at entry and weeks 24, 48, and 72 in real time at the local laboratory. Insulin will be batched and analyzed at the end of the study.

Plasma HIV-1 RNA
HIV-1 RNA must be performed by a laboratory that possesses a CLIA certification or equivalent. Eligibility will be determined based on the screening value.

CD4+ Count
Obtain absolute CD4+/CD8+ counts and percentages. All laboratories must possess a CLIA certification or equivalent.

Safety Labs
CBC with differential, chemistry panel including liver enzymes (ALT, AST, Alk Phos), total bilirubin, serum creatinine, and pregnancy test. All safety labs, HIV-1 RNA, and CD4 cell counts will be done at local certified laboratories.

Fasting Stored Serum, Plasma and Urine

If subjects come to the appointment and are not fasting, they will need to come back in fasting state for these evaluations within the 7-day window.

Serum (3 mL), plasma (3 mL), and clean-catch urine (minimum of two and maximum of six 3 mL aliquots. Please see Manual of Operations for further details) should be collected as indicated in the schedule of events (entry, week 24, week48, and week 72). Additionally, please collect 3ml of both serum and plasma in the event of study discontinuation and/or virologic failure. Frozen specimens will be collected, labeled with patients' PID and date of draw, and stored in a –70 degrees freezer (urine specimens may be refrigerated up to 24 hours prior to freezing if needed) until the study chairs inform the sites about the timing and location to which shipment should occur. The stored samples will be used for future measurements of inflammatory markers such as TNF-α, soluble TNF receptors I and II, IL-6, myeloperoxidase (MPO), prostaglandin metabolites, and cardiovascular and endothelial activation markers such as soluble intercellular adhesion molecule-1 (sICAM-1), soluble vascular cell adhesion molecule-1 (sVCAM-1), and von Willebrand factor (vWF).

7.0 Toxicity Management

Criteria for subject management, dose interruptions, modifications, and discontinuation in drug treatment will be mandated only for toxicities attributable to raltegravir. Toxicities due to drugs in the background regimen should be managed according to standard clinical practice, with the goal of maintaining continuous therapy, if possible.

7.1. Grade 1 or 2 Toxicity

Subjects who develop a Grade 1 or 2 AE or toxicity may continue raltegravir.

7.2 Grade 3 Toxicity

Subjects who develop a Grade 3 AE or toxicity should have all antiretroviral medications, including raltegravir, withheld unless the investigator has compelling evidence that the AE has NOT been caused by raltegravir. The study chairs (Drs. Currier, McComsey, or Lake) should be notified of any Grade 3 or greater toxicity. The subject should be re-evaluated closely until the AE returns to Grade ≤ 2, at which time raltegravir may be reintroduced at the discretion of the site investigator or according to standard practice.

If the same Grade 3 AE recurs within 4 weeks and it is thought to be related possibly, probably, or definitely to raltegravir, then raltegravir must be permanently discontinued. If the same Grade 3 AE recurs after 4 weeks but is not believed to be related to raltegravir, the management scheme outlined above may be repeated.

Subjects experiencing Grade 3 or greater AEs (requiring permanent discontinuation of raltegravir) should be followed closely for resolution of the AE to Grade ≤2, and the study chairs must be consulted.

Subjects with Grade 3 asymptomatic laboratory abnormalities in cholesterol, creatine kinase (CK), or triglycerides may continue raltegravir. CK is not a safety lab, and is to be checked only if the site PI feels it is warranted.

7.3 Grade 4 Toxicity

Subjects who develop a Grade 4 symptomatic AE or toxicity will have raltegravir discontinued. If the site investigator has compelling evidence that the AE has not been caused by raltegravir, dosing may resume when the AE has resolved and after consulting with the study chairs. Subjects

experiencing Grade 4 AEs requiring permanent discontinuation of raltegravir should be followed closely until resolution of the AE to Grade ≤2, and the study team must be consulted.

Subjects with Grade 4 asymptomatic laboratory abnormalities in cholesterol, CK, or triglycerides may continue raltegravir.

8.0 Criteria For Discontinuation

8.1 Permanent Study Drug Discontinuation

Study drug discontinuation can occur for the following reasons:

- Drug-related toxicity requiring permanent discontinuation (see Section 7.1).
- Requirement for prohibited concomitant drugs (see section 5.4).
- Request by subject to terminate treatment.
- Confirmation of virologic failure (two measurements of HIV RNA > 50 copies 2 weeks apart).
- Clinical reasons believed life-threatening by the physician, even if not addressed in the toxicity section of the protocol.
- Subject repeatedly noncompliant with raltegravir as prescribed.
- Subject discontinues raltegravir for ≥14 consecutive days.
- Subject discontinues background entry regimen for ≥14 consecutive days.
- Failure by the subject to attend ≥3 consecutive clinic visits.
- Pregnancy or breast-feeding.

8.2 Premature Study Discontinuation

- Request by the subject to withdraw.
- Request of the primary care provider if s/he thinks the study is no longer in the best interest of the subject.
- Subject judged by the investigator to be at significant risk of causing harm to self.
- At the discretion of the IRB, Food and Drug Administration (FDA), Health Canada, Office for Human Research Protections (OHRP), investigator, or the pharmaceutical sponsor Merck.

8.3 Early Discontinuation

Any subject who develops a Grade 3 or 4 symptom or laboratory abnormality (other than CK, bilirubin, cholesterol, and triglycerides, UNLESS THE INVESTIGATOR HAS A COMPELLING REASON TO THINK IT IS NOT RELATED TO RALTEGRAVIR) will be evaluated and will have study treatment withheld until the toxicity resolves (< grade 2). If the abnormality is thought to be related to study treatment, the subject will not be rechallenged without discussion with protocol chairs (see Section 7.2). Subjects who have to discontinue study treatment early (for the above listed reasons or confirmed virologic failure) will undergo a final safety evaluation and CT scan. Serum and plasma will also be banked at the time of study discontinuation (in lieu of the 24 or 48 week sample). All subjects will remain in follow-up for resolution of grade 3 or 4 toxicities.

9.0 Sample Size and Endpoints

9.1 Sample size

In a recently reported study of growth hormone releasing factor in HIV lipodystrophy (Falutz 2007), the FDA approved the use of 8% change in visceral abdominal fat between the active and placebo arms to define a meaningful difference in visceral abdominal fat. However, we will aim for 10% difference in visceral fat to make it more clinically significant.

We estimate that a sample size of 18 women per arm will provide > 80% power to detect a 10% difference in VAT at 24 weeks between the raltegravir-treated patients and those who remain on their current ART. We have increased the sample size to 20 women per arm to improve our power to look at secondary endpoints, and to account for potential loss of follow up. The total sample size is 40 women.

9.2 Randomization and Stratification

Subjects will be randomized with equal probability to the two arms of the study. Stratification will be by current ART regimen (PI- or NNRTI-containing).

9.3 Primary Endpoint Assessments

VAT volume (cm2) will be assessed by CT scan from a single 5 mm slice obtained at the level of the L4-L5 inter-vertebral disc space at weeks 0, 24, 48, and 72. All CT scans will be standardized and communicated via electronic medium (CD, DVD, or optic disk). Central reading will be performed at Tufts University. The central reader will be blinded to the patients' characteristics and study arm assignment.

9.4 Secondary Endpoints Assessments

- Abdominal SAT and VAT:SAT ratio, as measured by CT scan
- Total cholesterol/HDL cholesterol ratio, triglycerides, LDL cholesterol (direct)
- Subject-reported outcomes related to body image
- HsCRP
- Insulin, glucose, HOMA-IR
- Frequency of both new onset depression or an improvement or worsening of ongoing depression.

9.5 Study Monitoring

This is an open label study. Accrual and a summary of all Grade ≥2 signs and symptoms and all Grade ≥3 laboratory abnormalities will be prepared by the central data management site at UCLA, and reviewed by the team monthly. The AE summary will be divided by treatment arms and reviewed regularly by the team, as will baseline characteristics, early treatment discontinuations, and study discontinuations. The data management center at UCLA will also prepare a quarterly report of all AEs by treatment arm to be reviewed by the team.

Approximately one year after enrollment of the first subject, an interim review of the study will occur. An independent Study Monitoring Committee (SMC) comprised of three HIV investigators not involved in the study will review accrual, AE summaries, off-treatment, and off-study rates broken down by randomized treatment arm. A SMC may also be convened if a reason is identified by the study chairs, the data management center at UCLA, or in consultation with the team.

9.6 Analyses

All statistical tests will be two-sided with nominal level of 0.05. Analysis will be exploratory without adjusting for multiple testing.

9.6.1 Primary Analysis

Comparison between treatment arms of the primary endpoint will be undertaken using the Wilcoxon rank-sum test. The primary analysis will be as-treated, excluding subjects who do not remain on the study regimen and/or subjects who do not have an observed primary endpoint. Subjects who discontinue raltegravir or background entry regimen for ≥ 14 consecutive days will no longer contribute data to the primary outcome after the date on

which they first discontinued medication. An intent-to-treat analysis will also be performed as a supplement.

9.6.2 Occurrence of Study-Related AE
The number of subjects who experience treatment-related AE from the first day of study treatment to Week 24 and from Week 24 to Week 48 will be reported by arm.

9.6.3 Proportion of Subjects who Discontinue Study Treatment
Proportion of subjects who discontinue the study treatment prior to Weeks 24 and 48 will be summarized by arm. Reasons for discontinuing study drug will be listed.

10.0 Data Collection, Monitoring, and Adverse Event Reporting

10.1 Records to Be Kept
Data will be collected onto Case Report Forms (CRFs) provided by the Data Management Center at UCLA, and will be labeled only with a subject's study ID number. CRFs will be stored in a locked cabinet available only to study personnel for a period of time determined by pertinent policies and regulations, and for at least two years after study discontinuation.

10.2 Role of Data Management
Instructions concerning the recording of study data on CRFs will be provided by the data management center. Each site is responsible for keying the data in a timely fashion within 14 days.

10.3 Clinical Site Monitoring and Record Availability
Heather McCreath from the UCLA Data Management Center will visit participating CRSs to review the individual subject records, including consent forms, CRFs, supporting data, laboratory specimen records, and medical records (physicians' progress notes, nurses' notes, individuals' hospital charts) to ensure protection of study subjects, compliance with the protocol, and accuracy and completeness of records. The monitor also will inspect sites' regulatory files to ensure that regulatory requirements are being followed and sites' pharmacies, drug storage, and dispensing facilities to review product storage and management.

10.4 Serious Adverse Event Reporting
The serious adverse event (SAE) reporting requirements and definitions for this study and the methods for SAEs to the Regulatory Center at University of California at Los Angeles (UCLA) are defined in the Merck Manual for SAE Reporting of Adverse Events to UCLA, dated 5/1/2008. The SAE Manual is included in the MOP and available on the UCLA Raltegravir in Women Web site: http://www.uclacarecenter.org/iisp2.html.

The study agent for SAE reporting is raltegravir.

SAEs must be documented on the Serious Adverse Event Reporting form (SAE Reporting Form) available in the MOP and on the Raltegravir in Women Web site: http://www.uclacarecenter.org/iisp2.html

The study chairs (Drs. Currier, McComsey, or Lake) must be notified for any Grade 3 or greater toxicity that does not have a clearly documented etiology not related to raltegravir.

All Grade 4 events, death, persistent or significant disability/incapacity, hospitalization or prolongation of hospitalization, or event that the site investigator deems medically significant and thought to be related possibly, probably, or definitely related to raltegravir must be reported by completing and faxing the SAE Reporting form to (310) 557-1899.

Serious Adverse Events must be reported during the protocol-defined SAE Reporting period, which is from enrollment until eight weeks following the subject's last dose of study drug.

After the end of the protocol-defined SAE Reporting Period stated above, sites must report serious, unexpected, clinically-suspected adverse drug reactions if the study site staff becomes aware of the event on a passive basis, i.e., from publicly available information.

All confirmed virologic failures will reported immediately to the DSMB. The DSMB will also receive quarterly SAE reports for review.

11.0 Human Subjects

11.1 Institutional Review Board (IRB) Review and Informed Consent

This protocol, the informed consent document (Appendix II), and any subsequent modifications will be reviewed and approved by the IRB or ethics committee responsible for oversight of the study. A signed consent form will be obtained from the subject prior to screening and prior to study continuation should procotol modifications be required that led to the development of an amended informed consent document. The consent form will describe the purpose of the study, the procedures to be followed, and the risks and benefits of participation. A copy of the signed consent form will be given to the subject, and this fact will be documented in the subject's record.

11.2 Subject Confidentiality

All laboratory specimens, evaluation forms, reports, and other records that leave the site will be identified by coded number only to maintain subject confidentiality. All records will be kept locked. All computer entry and networking programs will be done with coded numbers only. Clinical information will not be released without written permission of the subject, except as necessary for monitoring by IRB, DSMB, FDA, Health Canada, the OHRP, or the pharmaceutical sponsor or their designee.

11.3 Study Discontinuation

The study may be discontinued at any time by the IRB, the pharmaceutical supporter, the FDA, Health Canada, the OHRP, or other government agencies as part of their duties to ensure that research subjects are protected.

12.0 Publication of Research Findings

Any presentation, abstract, or manuscript will be made available for review by the pharmaceutical sponsor prior to submission.

13.0 Biohazard Containment

As the transmission of HIV and other blood-borne pathogens can occur through contact with contaminated needles, blood, and blood products, appropriate blood and secretion precautions will be employed by all personnel in the drawing of blood and shipping and handling of all specimens for this study, as currently recommended by the Centers for Disease Control and Prevention and the National Institutes of Health.

All dangerous goods materials, including diagnostic specimens and infectious substances, must be transported according to the instructions detailed in the International Air Transport Association (IATA) Dangerous Goods Regulations.

14.0 Proposed Sites

All sites have experience conducting metabolic studies, HIV expertise and available populations of women. Each site will enroll 10women on average.

UCLA CARE Center (J.Lake and J.Currier)
Case Western Reserve (G. McComsey)
Tufts (C. Wanke)
Vanderbilt/Comprehensive Care Center (T. Hulgan)
University Health Network - Toronto General Hospital, Immunodeficiency Clinic (S. Walmsley)

15.0 References

1. Arribas J, Pozniak A, Gallant J, et al. Three-year safety and efficacy of emtricitabine (FT)/tenofovir DF (TDF) and efavirenz (EFV) compared to fixed dose zidovudine/lamivudine (CBV) in antiretroviral treatment-naïve patients [Abstract WEPEB029]. Fourth IAS Conference on HIV Pathogenesis, Treatment and Prevention, Sydney, 2007.

2. Bacchetti P, Gripshover B, Grunfeld C, et al. Fat distribution in men with HIV infection. J Acquir Immune Defic Syndr. Oct 1 2005;40(2):121-131.

3. Carr A. HIV lipodystrophy: risk factors, pathogenesis, diagnosis and management. AIDS. Apr 2003;17 Suppl 1:S141-148.

4. Carr A, Workman C, Smith DE, et al. Abacavir substitution for nucleoside analogs in patients with HIV lipoatrophy: a randomized trial. Jama. Jul 10 2002;288(2):207-215.

5. Cooper DA GJRJ, Katlama Cetal. Results of BENCHMRK-1, a Phase III Study Evaluating the Efficacy and Safety of MK-0518, a Novel HIV-1 Integrase Inhibitor, in Patients with Triple-class Resistant Virus. 14th Conference on Retroviruses and Opportunistic Infections February 25-28, 2007. Los Angeles, CA.

6. Falutz J, Allas S, Blot K, et al. Effects of TH9507, a Growth Hormone Releasing Factor Analog, on HIV-associated Abdominal Fat Accumulation: A Multicenter, Double-blind Placebo-controlled Trial with 412 Randomized Patients. 14th CROI, February 2007 Abstract #45.

7. Gervasoni C, Ridolfo AL, Trifiro G, et al. Redistribution of body fat in HIV-infected women undergoing combined antiretroviral therapy. AIDS. Mar 11 1999;13(4):465-471.

8. Grinsztejn B, Nguyen BY, Katlama C, et.al. Potent Antiretroviral Effect of MK-0518, a Novel HIV-1 Integrase Inhibitor, in Patients with Triple-class Resistant Virus. 46th Annual Interscience Conference on Antimicrobial Agents & Chemotherapy September 27-30, 2006. San Francisco, CA.

9. Haubrich RH, Riddler S, DiRienzo G, Komarow L, Powderly W, Garren K, George T, Rooney J, Mellors J, Havlir D, and the AIDS Clinical Trials Group 5142 Study Team. Metabolic Outcomes of ACTG 5142: A Prospective, Randomized, Phase III Trial of NRTI-, PI-, and NNRTI-sparing Regimens for Initial Treatment of HIV-1 Infection. Absract 38. The 14th Conference on Retroviruses and Opportunistic Infections February 25-28, 2007. Los Angeles, CA.

10. Markowitz M, Nguyen BY, Gotuzzo F, et.al. Potent Antiretroviral Effect of MK-0518, a Novel HIV-1 Integrase Inhibitor, as Part of a Combination ART in Treatment Naive HIV-1 Infected Patients. 16th International AIDS Conference August 13-18, 2006. Toronto, Canada.

11. Markowitz M, Morales-Ramirez JO, , Nguyen BY, et al. Antiretroviral activity, pharmacokinetics, and tolerability of MK-0518, a novel inhibitor of HIV-1 integrase, dosed as monotherapy for 10 days in treatment-naive HIV-1-infected individuals. JAIDS,2006 Dec 15;43(5):509-15.

12. Martin A, Smith DE, Carr A, et al. Reversibility of lipoatrophy in HIV-infected patients 2 years after switching from a thymidine analogue to abacavir: the MITOX Extension Study. AIDS. Apr 30 2004;18(7):1029-1036.

13. McComsey GA, Ward DJ, Hessenthaler SM, et al. Improvement in lipoatrophy associated with highly active antiretroviral therapy in human immunodeficiency virus-infected patients switched from stavudine to abacavir or zidovudine: the results of the TARHEEL study. Clin Infect Dis. Jan 15 2004;38(2):263-270.

14. Miller J, Carr A, Emery S, et al. HIV lipodystrophy: prevalence, severity and correlates of risk in Australia. HIV Med. Jul 2003;4(3):293-301.

15. Moyle GJ, Sabin CA, Cartledge J, et al. A randomized comparative trial of tenofovir DF or abacavir as replacement for a thymidine analogue in persons with lipoatrophy. AIDS. Oct 24 2006;20(16):2043-2050.

16. Saves M, Raffi F, Capeau J, et al. Factors related to lipodystrophy and metabolic alterations in patients with human immunodeficiency virus infection receiving highly active antiretroviral therapy. Clin Infect Dis. May 15 2002;34(10):1396-1405.

17. Steigbigel R Jetal JP et al. Results of BENCHMRK-2, a Phase III Study Evaluating the Efficacy and Safety of MK-0518, a Novel HIV-1 Integrase Inhibitor, in Patients with Triple-class Resistant Virus. 14th Conference on Retroviruses and Opportunistic Infections February 25-28, 2007. Los Angeles, CA.

18. Wanke C, Gerrior J, Kantaros J, Coakley E, Albrecht M. Recombinant human growth hormone improves the fat redistribution syndrome (lipodystrophy) in patients with HIV. AIDS. Oct 22 1999;13(15):2099-2103.

APPENDIX I
Data and Safety Monitoring Board members

Michael P. Dube, M.D.
Professor of Clinical Medicine
Department of Medicine, Division of Infectious Diseases
University of Southern California School of Medicine

Marshall J. Glesby, MD, PhD
Associate Professor of Medicine and Public Health
Co-Director, Cornell Clinical Trials Unit
Division of International Medicine and Infectious Diseases
Weill Cornell Medical College

Kathleen Squires, M.D.
Professor of Medicine
Director, Division of Infectious Diseases and Environmental Medicine
Thomas Jefferson University

APPENDIX II

[CLINICAL TRIAL SITE]

CONSENT TO PARTICIPATE IN RESEARCH

Phase II Study of Raltegravir as Replacement for Protease Inhibitor- or Non-Nucleoside Reverse Transcriptase Inhibitor-Based Antiretroviral Therapy in Women with Fat Accumulation

Lay language title: Women, Integrase, and Fat Accumulation Trial

INTRODUCTION

You are being invited to participate in a research study because you are a woman who is receiving treatment for HIV and have increased abdominal fat. The following information is provided in order to help you make an informed decision whether or not to participate in this study. This is a consent form. It gives you information about this study. You should review this information and ask questions about anything you do not understand. The study staff will talk with you about this information. You are free to ask questions about this study at any time. If you agree to take part in this study, you will be asked to sign this consent form. You will get a signed and dated copy to keep. Your participation in this study is voluntary. You may decide not to take part or to withdraw from the study at any time without losing the benefits to your routine medical care.

This is a research study to see if there is less fat accumulation in women who take raltegravir. We will be looking at fat accumulation or reduction of fat in women who are on a standard regimen of effective antiretroviral therapy compared to women who switch to a raltegravir-based regimen. We will be using a CT scan to determine this comparison. The study drug is made by and this trial is sponsored by Merck & Co, Inc.

If you receive your HIV care outside the UCLA CARE Center, it is important that your primary HIV care provider know that you are taking part in this study. Your study doctor will communicate with your primary HIV care provider to ensure coordination of your HIV care during your participation in this study.

Your participation in this study involves 13 visits over a period of about 72 weeks. This study will involve approximately 40 people.

DISCLOSURE

Your health care provider may be an investigator for this study, and as an investigator, is interested both in your clinical welfare and in the conduct of this study. Before entering this study or at any time during the research, you may ask for a second opinion about your care from another doctor who is in no way associated with this project. You are not under any obligation to participate in any research project offered by your doctor. The study doctor will be paid by Merck & Co, Inc., for conducting this study.

The study is sponsored by Merck & Co., Inc. The Principal Investigator of this study, Dr. Judith Currier, received an honorarium from Merck & Co., Inc. in the amount of $1,050 for participation in three Advisory Board teleconferences in the past year.

PROCEDURES

There are several procedures that you will be asked to undergo if you agree to take part in the study and sign this consent form. These are described below and in a chart at the end of this form. **All procedures are being done for research purposes only.**

The study will consist of 13 visits to the study doctor over the period of approximately 72 weeks.

Visit 1 - Screening

After obtaining informed consent a series of baseline evaluations will be performed.

During this visit, you will be asked to report some personal information, such as whether or not you smoke tobacco, alcohol use, family history of illnesses, and any illnesses (including HIV-related illness) or surgeries you have had. You will be asked about any medications you take, including medicines for HIV. Body measurements including weight and measurements of your hips, waist, and neck will be collected. The research staff will complete a physical exam, take your height, vital signs (blood pressure, pulse, temperature, breathing rate) and take some blood to send to the laboratory for testing. About 4 teaspoons of blood will be taken to test for the following:

- safety labs (liver enzymes, blood chemistry and blood counts)
- HIV viral load

For women capable of having children, a urine or blood pregnancy test will be completed as part of the safety lab evaluation at this visit and all future visits. You must notify the research staff if you are pregnant, think you may be pregnant, or if you are trying to become pregnant.

You will also be asked to sign a release of medical records form so that we can obtain information regarding your past-HIV care from your primary doctor. The research staff must obtain this information to determine whether or not you are eligible to participate in the study.

Visit 2 Baseline (within 30 days of screening)
The research staff will review all the information from the previous visit and your medical records to ensure you are still able to participate in the study. The research staff will ask you about any medicine you are taking, and whether you have been sick since your last visit. **You will be asked to come in fasting (i.e. not having anything to eat or drink for 8 hours).** A physical exam will be completed that includes taking your blood pressure, temperature, breathing rate, and pulse. About 2 tablespoons of blood will be taken to test for the following:

- safety labs
-
- immune function
- fasting lipids (cholesterol, HDL, LDL, and triglyceride measurements) and insulin
- inflammatory markers

Body measurements, including height, weight, and measurements of your hips, waist, and neck will be collected.

At this visit, you will also be asked to complete 2 questionnaires in a private room. One questionnaire will ask you about your body image and your quality of life. The other questionnaire will ask you about your feelings. They will be multiple-choice. Please ask the study staff if you do not understand any of the questions. You have the right to refuse to answer any question that you do not wish to answer. The questionnaires will take approximately 25 minutes to complete.

To complete this visit, you will also be asked to undergo a CT scan. A CT scan (computerized tomography) is a picture of a cross section of your body taken by a machine that uses advanced x-ray technology. In this study, the CT scanner will take an image of your body at the abdominal level in order to measure the amount of abdominal fat. A CT will be also required at weeks 24, 48, and 72 (the post-study follow-up visit).

At this visit you will be placed into one of two arms of the study, Arm A or Arm B. You have a 50/50 chance of being in either arm.

ARM A: Immediate switch: Switch PI or NNRTI to raltegravir
ARM B: Continue current therapy unchanged for 24 weeks then switch PI or NNRTI to raltegravir

After you receive your first dose of raltegravir, you will need to bring with you to every visit any remaining doses of raltegravir.

Subjects will be offered to participate in an optional photography portion of the study at this time. Photographs will be taken at baseline, Week 24, and Week 48. Only the study staff, the study physician, and perhaps other physicians involved in the study will be authorized to view the photographs. No photographs of the your face will be taken at any time.

Visit 3 – Week 4
During this visit you will have a targeted physical exam with weight and blood pressure and tests for viral load and safety labs. For these two tests, approximately 4 teaspoons of blood will be drawn.

Visit 4 – Week 8
During this visit you will have a targeted physical exam with weight and blood pressure and tests for viral load and safety labs. For these two tests, approximately teaspoons of blood will be drawn.

Visit 5 – Week 12
During this visit you will have a targeted physical exam with weight and blood pressure and tests for viral load, CD4 cell counts and safety labs. For these three tests, approximately 4 teaspoons of blood will be drawn.

Visit 6 – Week 18

During this visit you will have a targeted physical exam with weight and blood pressure and a test for safety labs. For this test, approximately 3 teaspoons of blood will be drawn.

Visit 7 – Week 24
During this visit you will have a targeted physical exam with weight and blood pressure. **You will be asked to come in fasting (i.e. not having anything to eat or drink for 8 hours).** Your waist and neck circumference and waist-to-hip ratio will be measured again. In addition, you will have tests for viral load, CD4 cell counts, fasting lipids, banked serum, and safety labs. For these tests, approximately 2 tablespoons of blood will be drawn.

At this visit, you will also be asked to complete 2 questionnaires in a private room. One questionnaire will ask you about your body image and quality of life. The other questionnaire will ask you about your feelings. They will be multiple-choice. Please ask the study staff if you do not understand any of the questions. You have the right to refuse to answer any question that you may not wish to answer. The questionnaires will take approximately 25 minutes to complete.

Your study doctor or study staff will schedule a CT scan to be taken at the [location] at your convenience within 14 days before or after this visit.

Visit 8 – Week 28
During this visit you will have a targeted physical exam with weight and blood pressure and a test for safety labs and viral load. For this test, approximately 4 teaspoons of blood will be drawn.

Visit 9 – Week 32
During this visit you will have a targeted physical exam with weight and blood pressure and a test for safety labs and viral load. For this test, approximately 4 teaspoons of blood will be drawn.

Visit 10 – Week 36
During this visit you will have a targeted physical exam with weight and blood pressure and tests for viral load and safety labs. For these two tests, approximately 4 teaspoons of blood will be drawn.

Visit 11 – Week 42
During this visit you will have a targeted physical exam with weight and blood pressure and a test for safety labs. For this test, approximately 3 teaspoons of blood will be drawn.

Visit 12 – Week 48
You will be asked to come in fasting (i.e. not having anything to eat or drink for 8 hours). During this visit you will have a targeted physical exam with weight and blood pressure and tests for viral load, CD4 cell counts, fasting lipids, banked serum, and safety labs. For these tests, approximately 2 tablespoons of blood will be drawn. Your waist circumference and waist-to-hip ratio will be measured again.

At this visit, you will also be asked to complete 2 questionnaires in a private room. One questionnaire will ask you about your body image and quality of life. The other will ask you about your feelings. They will be multiple-choice. Please ask the study staff if you do not understand any

of the questions. You have the right to refuse to answer any question that you may not wish to answer. The questionnaires will take approximately 25 minutes to complete.

Your study doctor or study staff will schedule a CT scan to be taken at the [location] at your convenience within 14 days before or after this visit.

At this visit you will either remain on raltegravir or you may switch to another regimen that you have discussed with your primary care provider. If you choose to continue taking raltegravir, this will be provided to you by the study. If you choose to change your anti-HIV regimen, you or your insurance company will be responsible for paying for this regimen.

Visit 13 – Week 72 (post-intervention follow-up)
The purpose of this visit is to see how you have been doing. You may still be taking raltegravir, or you and your doctor may have changed your medicines. Either way, your attendance at this visit is important.

You will be asked to come in fasting (i.e. not having anything to eat or drink for 8 hours). During this visit you will have a targeted physical exam with weight and blood pressure and tests for viral load, CD4 cell counts, fasting lipids, banked serum, and safety labs. For these tests, approximately 2 tablespoons of blood will be drawn. Your waist circumference and waist-to-hip ratio will be measured again.

At this visit, you will also be asked to complete 2 questionnaires in a private room. One questionnaire will ask you about your body image and quality of life. The other will ask you about your feelings. They will be multiple-choice. Please ask the study staff if you do not understand any of the questions. You have the right to refuse to answer any question that you may not wish to answer. The questionnaires will take approximately 25 minutes to complete.

Your study doctor or study staff will schedule a CT scan to be taken at the [location] at your convenience within 14 days before or after this visit.

Premature Study Treatment/Background ART Discontinuation Evaluations
If you discontinue raltegravir due to an adverse event, you will be followed to make sure your event is resolved. If you discontinue for any reason we would like for you to complete the week 12 and 24 evaluations, as listed in the Schedule of Events, before you are taken off study.

All samples will be destroyed at the end of this study.

POTENTIAL RISKS AND DISCOMFORTS

Side effects are unpleasant feelings or conditions that people get when they take a medication. Most medications cause side effects. Many of these side effects do not happen very often. It is also possible that you may have no side effects at all. Even so, you should know what to expect from the medication that will be used in this study.

Any side effect, including changes in medical conditions you had when you started the study should be reported to the study doctor. In addition, if you need to take any new medications or change the dose for a current medication during the study, you should report this to the study doctor.

It is important for your safety that you are completely truthful with the study staff about your health history, how you feel, and the medications that you are taking.

Risks of Raltegravir

Raltegravir has been given to patients in 5 different studies that are taking place now. In every patient study, raltegravir is given along with other HIV drugs. In 2 studies, the patients have never taken HIV drugs before. In the other 3 studies, the patients were on HIV drugs for a long time but their HIV drugs were not working. In 2 of the 5 studies, it is known which patients got the study drug or placebo. There are over 300 patients getting raltegravir in these two studies. It is estimated that about 500 patients are getting raltegravir in the other 3 studies. The longest that anyone has taken raltegravir is about 1.5 years. Raltegravir has also been given for shorter time periods to about 221 volunteers in 17 studies.

<u>In the two studies where it is known which patients got raltegravir or placebo:</u>

Some rare serious side effects (each happened in only 1 patient) judged as possibly related to raltegravir or other HIV drugs given along with raltegravir are:

- Inflamed pancreas (pancreatitis)
- Changes in blood chemistry (metabolic acidosis), decrease in kidney function and death

Less common side effects (occurred in more than 2 but less than 5 out of 100 patients in either study) that were judged to be related to raltegravir, given along with other HIV drugs are:

- Itching
- Feeling very tired
- Vomiting
- Altered taste
- Constipation
- Uneven distribution of body fat
- Sleepiness
- Abdominal pain
- Loss of appetite
- Muscle spasms
- Rash

Abnormal blood tests judged by the study doctor to be a side effect (occurred in more than 2 but less than 5 out of 100 patients in either study) of raltegravir, given along with other HIV drugs are:

- Increased liver function tests which may be a sign of liver problems
- Increase in an enzyme associated with muscle tissue which may be a sign of problems with the kidneys or heart
- Increase in an enzyme that helps digest fats

There were other abnormal blood tests that were not judged to be side effects by the study doctors but that caused patients to stop the study drug. Most of these abnormal test results improved and most patients were able to continue taking the study drug. In these studies, similar side effects were seen in the patients who received placeboalong with their other HIV drugs.

The most common side effects (occurred in at least 5 out of 100 patients in either study) that were judged to be related to raltegravir, given along with other HIV drugs are:

- Nausea
- Headache
- Dizziness
- Diarrhea
- Insomnia
- Abnormal dreams
- Intestinal gas

In the three studies where it is not yet known which patients received raltegravir or placebo:

Rare serious side effects (each happened in only 1 patient) judged related to the use of study drug, given along with other HIV drugs. These side effects are:

- Difficulty breathing
- Bone pain
- Rash
- Increase in blood sugar
- Fever
- Irritation of the stomach lining (gastritis)

The most common side effects (occurred in at least 5 out of 100 patients in at least one of the studies) judged related to the use of study drug given along with other HIV drugs are:

- Diarrhea
- Nausea

A greater incidence of cancer has been observed in subjects taking raltegravir than in people not taking raltegravir. **It is not believed that raltegravir caused the cancers to occur.**

Rarely, worsening of depression has been reported in patients switched to raltegravir. It is not yet known whether these mood symptoms were due to raltegravir. During the course of the study, you will be monitored closely for signs and symptoms of depression.

Recently, data was made available from another study sponsored by Merck and Company. The purpose of that study was to compare cholesterol profiles in men and women who stayed on some of their HIV medications but switched from Kaletra (a protease inhibitor) to raltegravir. In that study, people who switched to raltegravir had a significant improvement in their cholesterol at 6 months. However, HIV viral loads went up in a few more patients who switched to raltegravir compared to those who stayed on Kaletra, and the study was stopped so that the investigators could find out why. We still do not know for sure why the viral loads for some people in the raltegravir group of that study went up. 66% of the patients whose viral loads went up reported that they had

had similar problems with other medications. After their viral loads went up, the viruses from those patients were analyzed. Many of them had resistance to multiple drugs. In fact, all of the patients had resistance to medications other than raltegravir, and only some had evidence of resistance to raltegravir. This suggests that patients viral loads might have gone up because the other medications they were taking were not able to help raltegravir keep the viral load undetectable. Also, some patients already had detectable viral loads when they entered the study. Therefore (although we do not know for sure), when viral loads went up in these patients, it may have had nothing to do with raltegravir.

The problem of viral loads going up in patients on raltegravir has not been seen in any other study. Still, to ensure your safety, you will not be allowed to participate in this study if you have had your viral load go up in the past while you were on medications, or if you have known resistance to any of the medicines you are currently taking.

There was no increased risk of a patient's viral load going up for women in that study, although women only comprised about 150 of the 700 patients participating. As mentioned, both men and women in that study had significant improvements in their cholesterol levels.

Risks of CT Scan

We are exposed to radiation on a daily basis both from natural (sun and earth) and human-made sources. In addition to the radiation that you may be exposed to as part of your clinical care (if you are receiving clinical care), you will receive **four CT scans** while participating in this research study.

The estimated radiation dose that you will receive as a result of the additional **four CT scans is 3000 millirem**, or **60%** of the 5,000 millirem **annual** limit allowed radiation workers.

Risks of Blood Drawing

Taking blood may cause pain, discomfort, bleeding, or bruising where the needle enters the body and, in rare cases, fainting, infection or blood clots.

Risks of Fasting

You may experience lightheadedness or nausea from fasting.

In addition, there may be discomforts or risks to you which are presently not foreseeable.

ANTICIPATED BENEFITS TO SUBJECTS

During this study, your condition will be monitored closely. You may receive no benefit by participating in this study.

ANTICIPATED BENEFITS TO SOCIETY

Information gained about the safety and effectiveness of the study drug may benefit patients in the future.

ALTERNATIVES TO PARTICIPATION

If you decide not to participate in this study, there are a number of approved treatments that are known to be effective in people with HIV infection. Your doctor will be able to tell you which of these approved treatments might benefit you. Alternatives to participation include both these approved treatments, and enrollment in other experimental protocols. There are no approved medications for the treatment of lipohypertrophy in HIV patients. Raltegravir is FDA-approved and may be available from your health care provider outside of this study. There may also be other experimental drugs available to you by enrolling in another study. Additionally, you may decide not to receive any treatment for your HIV at this time.

PAYMENT FOR PARTICIPATION

For your time and to help reimburse you for your expenses, you will receive $[payment amount determined by each site] for each clinic visit.

If you decide to leave the study or if you are discontinued for any reason, you will be reimbursed for all study days you have participated in. Total possible payment for your completion of the entire study is [amount].

FINANCIAL OBLIGATION

There will be no cost to you for study drug, clinic visits, examinations, or laboratory and test procedures that are part of this study. You or your insurance provider should continue to pay for expenses for your current medical care and prescriptions. You or your insurance provider may also be responsible for the regimen of approved anti-HIV medications that is best for you.

It is possible that your insurance will not pay for all the treatments and tests you will receive if you participate in this research. That is because many insurance companies, HMOs and health benefits plans do not cover the cost of standard treatments that are provided as part of a research study. If that happens, you will be responsible for all charges related to your treatment including cost of the medication, pharmacy dispensing fees, office visits, laboratory charges, radiological studies, and hospitalization charges (if necessary). The study doctor will provide you with an estimate of the cost of participation in the research, if applicable.

EMERGENCY CARE AND COMPENSATION FOR INJURY

If you are injured as a direct result of research procedures, you will receive treatment at no cost. The University of California and Merck & Co., Inc. do not provide any other form of compensation for injury.

PRIVACY AND CONFIDENTIALITY

The only people who will know that you are a research subject are members of the research team and, if appropriate, your physicians and nurses. No information about you, or provided by you during the research, will be disclosed to others without your written permission, except if necessary to protect your rights or welfare (for example, if you are injured and need emergency care) or if required by law. People who may review your records include: Merck & Co., Inc., the UCLA Office for Protection of Research Subjects, and their designees. As a result, they may see your name, but they are bound by the rules of confidentiality not to reveal your identity to others.

When the results of the research are published or discussed in conferences, no information will be included that would reveal your identity.

Authorized representatives of the Food and Drug Administration (FDA), the study sponsor and those working for the sponsor, independent ethics committees, and inspectors from foreign government regulatory agencies may need to review records of individual subjects. However, no representative may take away any identifying information about you from the clinic site (e.g., names, addresses). As a result, they may see your name, but they are bound by rules of confidentiality not to reveal your identity to others.

There is a second, more detailed explanation of how health data about you will be used and shared with others that is required by federal law, i.e., Health Insurance Portability and Accountability Act. This consent is a separate form. If you decide not to sign this separate form, you will not be allowed to participate in this research study.

PARTICIPATION AND WITHDRAWAL

Your participation in this research is VOLUNTARY. If you choose not to participate, that will not affect your relationship with UCLA (or UCLA Medical Center), or your right to health care or other services to which you are otherwise entitled. If you decide to participate, you are free to withdraw your consent and discontinue participation at any time without prejudice to future care at UCLA.

WITHDRAWAL OF PARTICIPATION BY THE INVESTIGATOR

Your participation in this study may be stopped at any time without you being asked. Your participation in the study may be stopped for any of the following reasons:

- The investigator decides it is in the best interest of your health and welfare
- You are unable to follow the investigator’s instructions
- If the sponsor, the FDA, or UCLA stops the study
- Administrative reasons
- If you become pregnant
- Some subjects may be discontinued from the study because of a lack or a loss of response to the treatment (for example, if your viral load goes up).
- If you receive additional HIV medications that are not prescribed by your study doctor.

NEW FINDINGS

During the course of the study, you will be informed of any significant new findings, good or bad, such as changes in the risks or benefits resulting from participation in the research or new alternatives to participation that might cause you to change your mind about continuing in the study. If new information is provided to you, your consent to continue participating in this study will be re-obtained.

IDENTIFICATION OF INVESTIGATORS

In the event of a research related injury or if you experience an adverse reaction, please immediately contact one of the investigators listed below. If you have any questions about the research, please feel free to contact:
[Names of study faculty/staff]

RIGHTS OF RESEARCH SUBJECTS

You may withdraw your consent at any time and discontinue participation without penalty. You are not waiving any legal claims, rights, or remedies because of your participation in this research study. If you have questions regarding your rights as a research subject, you may contact the Office for Protection of Research Subjects, [address and phone number]

SIGNATURE OF RESEARCH SUBJECT

I have read the information provided in this consent form. I have been given an opportunity to ask questions and all of my questions have been answered to my satisfaction. I have been given a signed and dated copy of this form, as well as a copy of the Subject's Bill of Rights.

BY SIGNING THIS FORM, I WILLINGLY AGREE TO PARTICIPATE IN THE RESEARCH IT DESCRIBES.

____
Name of Subject

______________________________________ ______________
Signature of Subject Date

SIGNATURE OF INVESTIGATOR

I have explained the research to the subject and answered all of his/her questions. I believe that he/she understands the information described in this document and freely consents to participate.

____
Name of Investigator

______________________________________ ____________________________
Signature of Investigator Date (must be the same as subject)

CHART OF PROCEDURES

	Screening	Baseline (Day 1)	Week 4	Week 8	Week 12	Week 18	Week 24	Week 28	Week 32	Week 36	Week 42	Week 48	Week 72
Informed Consent	X												
Medical History	X												
Targeted physical exam		X	X	X	X	X	X	X	X	X	X	X	X
Complete physical exam	X												
Fasting for 8 hours		X					X					X	X
Body image/quality of life questionnaire		X					X					X	X
Depression screening		X					X					X	X
CT scan		X					X					X	X
Blood for safety	X	X	X	X	X	X	X	X	X	X	X	X	X
Fasting Lipids		X					X					X	X
Pregnancy test*	X	X	X	X	X	X	X	X	X	X	X	X	X
Blood for HIV viral load	X		X	X	X		X	X	X	X		X	X
Blood for CD4 cell count		X			X		X					X	X
Waist and neck circumference and waist-to-hip ratio		X					X					X	X
Urine samples		X					X					X	X

* If you are a woman who is able to become pregnant.

APPENDIX III – CLINICAL TRIALS PROTOCOL REGISTRATION #
NCT00656175

APPENDIX IV – Consent for Photography

CARE 001 ADDENDUM: PHOTOGRAPHY CONSENT FORM

Lay Language Title: Women, Integrase, and Fat Accumulation Trial

Protocol Title: Phase II Study of Raltegravir as Replacement for PI- or NNRTI-Based ART in Women with Fat Accumulation

INTRODUCTION

As a subject in the CARE 001 research study, you previously reviewed and signed an informed consent document for the main study. This form is an addendum to the full consent form, and will provide you the option to participate in a portion of the study where we try to document changes in fat distribution by photographing the areas of your body which are most affected. Participation in this portion of the study is completely voluntary.

The information below explains the reasons why we feel photographs would help us in documenting fat distribution changes, the risks of participation, and our expectations of you as a subject. After reading this consent form, please ask questions about anything you do not understand or would like more information about. After reading this consent form and discussing the contents with your physician, if you wish to participate in this part of the study we will ask you to sign and date this form. After signing the consent form, you will be given a copy for your records.

Please remember that your participation is voluntary. If you choose to sign the consent form, you retain the right to withdraw your consent at any time. If you do not consent to photography, it will not change your ability to participate in the CARE 001 study or any other study.

PURPOSE OF THE STUDY

As stated in the full consent form, one goal of this study is to monitor changes in fat distribution across your body after switching from your old medication regimen to a raltegravir-containing regimen. Specifically, we want to measure whether there is normalization in areas where you previously gained or lost fat while on a protease inhibitor- (PI) or non-nucleoside (NNRTI)-containing regimen. In some subjects, this change may be subtle, or they may be no change at all. Other subjects may see and feel a significant change. Although we will be taking measurements of your body over the 48 weeks of the study, taking photographs of the changes in your body is an easy way not only to document these changes, but also to give a general sense of how your body is changing over time. These photographs are also useful for illustrating changes in fat redistribution in medical publications and reports. If your photographs are chosen to appear in the body of a scientific publication or presentation, your identity will not be revealed, and every effort will be made to maintain your privacy (as outlined below).

PROCEDURES

If you consent to participation in this part of the study, a set of photographs will be taken at four timepoints: baseline, 24 weeks, 48 weeks, and 72 weeks. Each set of photographs will only take a couple of minutes to perform. The areas to be

photographed may vary by individual, but will be the areas of your body which are most affected by changes in fat distribution. We will not photograph your face during any of these visits. In this way, your identity will be protected. The photographs will be stored in a password-protected file at your clinic. Only your study identification number will be tied to the photographs in an effort to further protect your privacy and prevent unauthorized persons from viewing these photographs. Only the study staff, your study physician, and, possibly, other physicians involved in the study, will be authorized to view your photographs.

POTENTIAL RISKS AND DISCOMFORTS

There are no foreseeable physical risks associated with the taking of these photographs. There are some psychological risks associated with this portion of the study. Some subjects feel a sense of embarrassment and/or being uncomfortable. We will try to minimize this risk as much as possible. A brassiere or sports bra should be worn for modesty. Unrestrictive pants that can be easily adjusted are recommended. No girdles may be worn. The study staff will adjust your clothing to allow maximum exposure of the areas to be photographed while making all attempts to preserve modesty. Only you and the study staff will be in the room at the time the photographs are being taken. If the study staff member seeing you on the day photographs are to be taken is male, a female staff member will accompany him. If you do not feel comfortable with a male staff member being in the room, you have the right to request that a substitute female staff member be found for that portion of the study visit. If at any point you feel excessively uncomfortable, you should notify a staff member.

ANTICIPATED BENEFITS TO SUBJECT

There are no direct benefits to the study participant of being photographed.

ANTICIPATED BENEFITS TO SOCIETY

Indirect benefits include the knowledge that the participant is aiding in medical education and research, and that her participation may promote an increased understanding of antiretroviral-associated lipodystrophy.

ALTERNATIVES TO PARTICIPATION

The alternative is not participating in this portion of the study. As mentioned, non-participation will not alter your ability to continue participating in the rest of the study.

PAYMENT FOR PARTICIPATION

You will receive no additional compensation for participating in this portion of the study. Additional compensation is also not provided should your photographs be chosen to appear in scientific publications or presentations.

FINANCIAL OBLIGATION

There is no cost to you to participate in this portion of the study.

EMERGENCY CARE AND COMPENSATION FOR INJURY

If you are injured as a direct result of research procedures not done primarily for your benefit, you will receive treatment at no cost. The University of California does not normally provide any other form of compensation for injury.

PRIVACY AND CONFIDENTIALITY

The only people who will know that you are a research subject are members of the research team and, if appropriate, your physicians and nurses. No information about you, or provided by you during the research will be disclosed to others without your written permission, except if necessary to protect your rights or welfare (for example, if you are injured and need emergency care).

Direct access to your medical records may be required by authorized people to check the information collected for the study. People who may review your records include: the U.S. Food and Drug Administration (FDA), the UCLA IRB (a committee that watches over the safety and rights of research subjects), National Institutes of Health (NIH), study staff, study monitors and their designees. As a result, they may see your name, but they are bound by the rules of confidentiality not to reveal your identity to others.

When the results of the research are published or discussed in conferences, no information will be included that would reveal your identity.

PARTICIPATION AND WITHDRAWAL

Your participation in this research is VOLUNTARY. If you choose not to participate, that will not affect your relationship with UCLA (or UCLA Medical Center), or your right to health care or other services to which you are otherwise entitled. If you choose not to participate, this will also not affect your participation in the main study. If you decide to participate, you are free to withdraw your consent and discontinue participation at any time without prejudice to future care at UCLA or your participation in the main study.

IDENTIFICATION OF INVESTIGATORS

Should you need to reach a study physician regarding an emergent matter or other question, the Principal Investigator and/or any of the Co-Investigators listed above may be contacted at:

RIGHTS OF RESEARCH SUBJECTS

As your participation is voluntary, you may withdraw your consent for participation in part or all of the study at any time without penalty or prejudice. Should you withdraw your consent, you do not forfeit your right to any applicable legal claims, rights, or remedies. If you have questions regarding your rights as a research subject, you may contact the UCLA Office for Protection of Research Subjects at: 11000 Kinross Building, UCLA, Box 951694, Los Angeles, CA 90095-1694, (310) 825-8714.

I have had the opportunity to read (or someone has read to me) the information provided to me in this consent form. I have been given the opportunity to ask questions and all of my questions have been answered to my satisfaction. I have been given a copy of this form.

BY SIGNING THIS FORM, I WILLINGLY AGREE TO PARTICIPATE IN THE RESEARCH DESCRIBED ABOVE.

____
Name of Subject

________________________________ ____________________________

Signature of Subject Date

Signature of Investigator

I have explained the information described in this document to the subject and answered all of her questions. I believe that she understands the information and freely consents to participate in this portion of the study.

____
Name of Investigator

____________________________ ____________________________
Signature of Investigator Date (must match the subject)
